# Healing and trauma services for Aboriginal and Torres Strait Islander people: a scoping review

**DOI:** 10.1080/00049530.2026.2678850

**Published:** 2026-06-11

**Authors:** Isabella Freijah, Rebekah Julian, Kelsey Madden, Kimberley A Jones, Amanda Jones, Helen Kennedy, Nina Levin, Shantai Croisdale, Catherine Chamberlain

**Affiliations:** a Indigenous Health Equity Unit, Melbourne School of Population and Global Health, University of Melbourne, Melbourne, Victoria, Australia; b Client Practice and Evidence Development, Victoria Aboriginal Child and Community Agency, Melbourne, Victoria, Australia

**Keywords:** Trauma-informed care, healing-informed care, Aboriginal and Torres Strait Islander people, social and emotional wellbeing, intergenerational trauma

## Abstract

**Objective:**

Colonisation and oppression of Aboriginal and Torres Strait Islander peoples have resulted in intergenerational trauma and systemic health inequities, making culturally responsive, healing-informed approaches that uphold self-determination essential. This review aimed to identify and describe healing services for Aboriginal and Torres Strait Islander people affected by trauma, violence or abuse.

**Method:**

A four-step search strategy located peer-reviewed studies, grey literature reports and organisational websites (2012-2025).

**Results:**

Eighty services/programs were identified across Australia with 15 exemplars included. Most were delivered by Aboriginal Community Controlled Organisations spanning justice, family violence prevention, primary health care, and community services. All exemplars included culturally grounded components such as yarning, storytelling, and time on Country. Evaluations suggested improvements in wellbeing, cultural connection and family and community relationships, though evaluation approaches varied. Synthesis of 31 healing frameworks produced an umbrella framework encompassing three guiding principles and eight service elements, including culturally centred, community-led, and holistic approaches. Exemplar services are aligned strongly with this framework.

**Conclusions:**

Healing-informed, culturally-grounded, community-led and flexible wrap-around approaches support wellbeing, cultural connection, and relationships for those affected by trauma, violence and abuse. Findings provide an evidence-informed framework for designing, delivering and evaluating future services, highlighting the need for Indigenous-led evaluation.

## KEY POINTS

**What is already known about this topic**:

(1) Aboriginal and Torres Strait Islander health and wellbeing is grounded in holistic social and emotional wellbeing frameworks that emphasise spirituality, kinship, community, culture and connection to Country.

(2) Colonisation, forced child removal, systemic racism and structural inequality have produced enduring intergenerational and collective trauma, contributing to persistent health inequities.

(3) Culturally responsive, community-led and healing-informed approaches are widely recognised as essential for improving wellbeing and addressing trauma among Aboriginal and Torres Strait Islander peoples.

**What this topic adds**:

(1) Demonstrates the importance of cultural determinants in service delivery for Aboriginal and Torres Strait Islander people affected by trauma, violence or abuse.

(2) Provides an evidence-informed conceptual framework for service delivery including three guiding principles and eight core service elements.

(3) Highlights gaps in evaluation evidence on services and the need for Indigenous-led evaluation methods.

## Introduction

Aboriginal and Torres Strait Islander communities represent some of the world’s oldest continuing cultures, dating back at least 65,000 years (Dudgeon et al., 2014). For thousands of years, Aboriginal and Torres Strait Islander communities have achieved holistic health and wellbeing through intricate systems of lore, storytelling, kinship, cultural traditions and a deep connection to Country (Langton & Corn, 2023). These enduring cultural systems continue to empower communities today, serving as vital sources of identity, strength and resilience (Bourke et al., 2018).

Health, as defined by Aboriginal and Torres Strait Islander people, encompasses social and emotional wellbeing (SEWB), which integrates spirituality, body, mind, emotions, kinship, community, culture and connection to Country (Gee et al., 2014). Strong connections between these domains protect health and wellbeing and foster resilience, whereas disruptions to these core connections can diminish an individual’s sense of self and wellbeing (Gee et al., 2014). For many Aboriginal and Torres Strait Islander peoples, the enduring legacy of colonisation combined with ongoing structural inequality has left deep and lasting emotional, social and health impacts (Menzies, 2019). This has been described as collective trauma (Krieg, 2009; Ratnavale, 2007) or intergenerational or transgenerational trauma (Atkinson, 2002).

Research has found that experiencing trauma is linked to an increased risk of poor health outcomes and subsequent health inequities for Aboriginal and Torres Strait Islander peoples (Nasir et al., 2021; Paradies, 2016; Sherwood, 2013). For example, members of the Stolen Generations over the age of 50 face higher rates of chronic illness and disadvantage (Australian Institute of Health and Welfare, 2021). These inequities extend across generations, with descendants also experiencing poorer health, higher rates of violence and incarceration, and other impacts from intergenerational trauma and systemic disadvantage (Australian Institute of Health and Welfare, 2021). Closing the gap in health outcomes between Aboriginal and Torres Strait Islander peoples and non-Indigenous Australians requires structural reforms that prioritise culturally responsive, healing-informed approaches. These approaches promote health equity, recognise the resilience and self-determination of Aboriginal and Torres Strait Islander peoples and create opportunities to regain a sense of control (Department of Health, 2021; Hinton et al., 2015). The community-controlled sector plays a vital role in this work, ensuring that programs and supports are developed for, with, and in response to the needs and aspirations of local communities. Community-led services and programs take a strength-based, holistic approach that reframes the role of culture and the legacy of colonialism in the delivery of health and wellbeing services (Dudgeon et al., 2022; Wise et al., 2024).

However, the 2021 Royal Commission into Victoria’s Mental Health System identified that existing services fail to meet the needs of Aboriginal and Torres Strait Islander people affected by trauma (Royal Commission into Victoria’s Mental Health System, 2021). Culturally responsive, community-led and healing-informed approaches are widely recognised as essential for addressing trauma and promoting wellbeing yet contemporary peer-reviewed evidence describing Australian-based healing services for this population is limited, outdated or focused on other populations or service types (Bulloch et al., 2019; Gupta et al., 2020; McKendrick et al., 2013; Wise et al., 2024; Yu et al., 2020).

In response, this scoping review aims to identify and describe healing services designed for Aboriginal and Torres Strait Islander people affected by trauma, violence or abuse. By systematically mapping available programs and services and synthesising guiding principles and core service elements, the review provides an evidence-informed framework to support culturally responsive and healing-informed services and programs for Aboriginal and Torres Strait Islander people affected by trauma, violence or abuse.

The review addressed the following research questions:

1. What services have been developed for Aboriginal and Torres Strait Islander people affected by trauma, violence or abuse?
2. What are the guiding principles or values underpinning these services?
3. What are the core components of these services?
4. Are there any key success factors underpinning delivery (e.g., effectiveness, acceptability and feasibility)?

### Author positionality

This review is Aboriginal-led, developed through a collaborative partnership between the Victorian Aboriginal Child and Community Agency (VACCA; AJ, HK, NL, SC) and the University of Melbourne (CC, IF, KJ, KM, RJ). Guided by Aboriginal leadership (CC, HK) and working with non-Indigenous allies (AJ, IF, KJ, KM, NL, RJ, SC), the review is grounded in cultural integrity, affirming our shared commitment to self-determination and driving meaningful, lasting change in the social and emotional wellbeing of Aboriginal and Torres Strait Islander peoples.

## Materials and methods

This review follows the Joanna Briggs Institute methodology for scoping reviews and adheres to the PRISMA-ScR checklist (Peters et al., 2020) and the CONSIDER statement (see Appendix 1 in Supplementary Materials; Huria et al., 2019).

### Eligibility criteria

The Population, Concept, Context framework was used to determine the eligibility criteria (see Table 1 and Table A1 in Appendix I).

**Table 1.** Inclusion and exclusion criteria.

Domain	Criteria
Participants	Aboriginal and Torres Strait Islander peoples. No exclusion criteria for age, gender or health status of participants.
Concept	A trauma-specific service or program designed to support healing or recovery for Indigenous peoples with experiences of trauma, violence and/or abuse. These include health services, programs, guidelines, models and frameworks. A service was defined as an organisational structure delivering trauma-related care or support. A program was defined as a set of activities or interventions delivered within a service or organisation.
Context	Trauma-specific services or programs for Indigenous peoples within a variety of settings including primary care, community-based care, school settings, Aboriginal Community-Controlled Organisations (ACCOs) and Aboriginal Community Controlled Health Organisations (ACCHOs).
Types of sources	Publications that provided a description and/or evaluation of a service or program that aimed to provide support for Aboriginal and Torres Strait Islander peoples with experiences of trauma, violence and/or abuse, including service evaluations, literature reviews, qualitative studies, governmental or organisational reports, websites and opinion pieces. Dissertations and conference abstracts were not eligible for inclusion.

### Search strategy

We used a four-step search strategy to locate peer-reviewed studies, grey literature reports and information from organisation websites:

1. We searched PsycINFO and Google Scholar for relevant articles. Title and abstract keywords, index terms and author expertise were used to develop the full search strategy.
2. We searched Medline (Ovid), EMBASE (Ovid), PsycINFO (Ovid), CINAHL (EBSCO) and Informit Indigenous Databases. The search strategy was designed around three core constructs: (1) trauma, violence and/or abuse; (2) service or program; and (3) Indigenous peoples. The final search strategy for each database is provided in Appendix 1.
3. A manual search was conducted of the HealthInfoNet website to identify and retrieve information about relevant services and programs.
4. Where further information was required, snowball searches were conducted using references of sources and service websites.

The search was restricted to papers written in English and published between January 2012 and July 2025. Although search terms included any Indigenous populations to identify internationally published literature, the search results were skewed towards Australian literature due to the inclusion of Aboriginal and Torres Strait Islander specific databases. As such, the inclusion criteria were adjusted to include Australian services and programs only.

### Evidence selection

Identified citations were exported into Endnote and then merged into Covidence (Veritas Health Innovation, n.d.) to remove duplicates. Pilot screening was undertaken, with all team members independently reviewing 10% of records to establish consensus on eligibility criteria. Three reviewers (IF, KM, RJ) then screened titles and abstracts, with a fourth reviewer (CC or KJ) adjudicating any disagreements. Another calibration exercise was conducted prior to full-text review, which was independently assessed by the same three reviewers, again with fourth reviewer adjudicating discrepancies. Records deemed ineligible for full-text screening were excluded and the reasons recorded.

Records deemed eligible for inclusion at full-text were categorised by their degree of relevance to the research questions and by the quality of evidence available (see Table A2 in Appendix 1). Highly relevant records with high-quality evaluation reports were selected as exemplar services or programs. This additional step was undertaken to distinguish between records with descriptive accounts of services and programs from records reporting detailed evaluation findings.

### Data charting

Following a pilot exercise conducted to calibrate a purpose-built data extraction form, data were extracted from eligible records by three independent reviewers (IF, KM, RJ). The data points of interest in *all* eligible records included service and/or program location, operational status (i.e., current or no longer delivered), target population, underpinning frameworks, guiding principles and core service components. Additional data on the evaluation indicators were extracted for the records of exemplar services or programs only. This included effectiveness, acceptability and feasibility (see Table A3 in Appendix 1 for further details). Data were extracted until saturation was reached and no new themes or components could be generated.

### Framework development

Healing frameworks identified through the search strategy (described above) were reviewed, and their guiding principles and service elements were extracted, mapped and compared in a spreadsheet to identify common and recurrent themes across frameworks. Overall, 31 healing frameworks were identified. Of these, four frameworks were identified that collectively encompassed the full range of guiding principles and service elements represented across the 31 frameworks (Commonwealth of Australia, 2017; The Healing Foundation, 2019, 2022; Hovane et al., 2023). These four frameworks were then reviewed by the authorship team, and consensus was reached that no additional guiding principles or service elements were required. The guiding principles and service elements from these four frameworks were subsequently synthesised to develop a consensus umbrella framework.

An overview of the umbrella framework is presented in Table 2. The framework comprises of three overarching guiding principles of healing and wellbeing services: acknowledge history and the impacts of colonisation; be trauma-aware and healing-informed; prioritise self-determination and empowerment. It also includes eight evidence-informed service elements: culturally centred; flexible, tailored responses for individuals and communities; community-led; safe; preventative, restorative, holistic and wraparound approaches; strengths-based; collaborative; accountable.

**Table 2.** Consensus umbrella framework.

Overarching guiding principles of healing and wellbeing services		
GP1	Acknowledge history and the impacts of colonisation	Healing supports Aboriginal peoples to overcome trauma and disadvantage. Intergenerational trauma is caused by the impacts of colonisation, loss, systemic barriers, and ongoing racism. These affect social and emotional wellbeing, health, income, personal safety, and justice
GP2	Be trauma-aware and healing-informed	Trauma-aware, healing informed practice ensures all initiatives to support healing are based on an understanding of the ongoing impacts of intergenerational trauma on individuals, families, and communities
GP3	Prioritise self-determination and empowerment	Healing will happen by enabling Aboriginal peoples to address distress, overcome trauma, strengthen connections, and restore wellbeing at the individual, family and community level. This is a continuous process throughout each person’s life and across generations
Evidence-informed service elements		
SE1	Culturally centred	To enhance a positive sense of identity, self-confidence, and hope Having a strong sense of identity through connection to culture, country, family, and community will help fulfil the cultural needs of individuals. It will help individuals and their families to know who they are, who they are connected to, and where they fit in the world
SE2	Flexible, tailored responses for individuals and communities	Recognition of cultural diversity: there is no one-size fits all approach as there is no single Aboriginal or Torres Strait Islander culture or group Healing services need to be locally defined, based on local lore, culture, knowledge systems, family and kinship systems and ways of working Services need to be flexible and transferable
SE3	Community-led	Aboriginal ownership of policies, programs, service design, implementation and evaluation across all systems, sectors, and organisations Local community at the front and centre of the service: “nothing about us, without us” Embedded in Indigenous led (community-controlled) organisations with demonstrated healing leadership or other culturally safe, trauma-informed organisations
SE4	Safe	Provision of culturally safe healing spaces which are spiritually, socially and emotionally safe, as well as physically safe for people Inclusive of physical elements that are culturally grounded Psychological safety for Aboriginal people through cultural respect and recognition
SE5	Preventative, restorative, holistic, wrap-around	Aboriginal people’s health is viewed in a holistic context that encompasses mental health and physical, cultural and spiritual health, including connection to land/country Interconnectedness between individual, family and community wellbeing, and the ripple effects of trauma Capacity building for individuals, families and communities Inclusive of practical supports (e.g., provision of food and supplies)
SE6	Strengths-based	Focus on the strengths that exist within their families and communities, which can provide the foundations for healing and pride in identity
SE7	Collaborative	Collaboration with community: to ensure readiness, capacity, local leadership Collaboration with local service sector through effective partnerships Two-ways thinking: combine western therapeutic support with Indigenous cultural healing
SE8	Accountable	Incorporate strong, culturally appropriate evaluation frameworks, communications plans and performance monitoring mechanisms to ensure responsibility, accountability, transparency and program sustainability

*Note*. GP = overarching guiding principle; SE = Evidence-informed service elements.

Of note, we use the term *trauma-aware, healing-informed* to reflect terminology used in Aboriginal and Torres Strait Islander health context, including those of the Healing Foundation and the National Aboriginal and Torres Strait Islander Health Plan 2021–2031 (Department of Health, 2021; Healing Foundation, 2020). While trauma-informed care emphasises recognising and responding to the impacts of trauma, healing-informed approaches additionally prioritise strengths-based healing and recovery. This framing shifts the focus from deficit-oriented understandings of trauma towards approaches that recognise resilience, cultural strength and the broader historical and structural contexts shaping experiences of trauma (Birnbaum, 2019; Haslam & McGrath, 2020; Smith & Monteux, 2023; Tseris, 2019).

### Quality assessment

An adapted general critical appraisal approach was used to assess the quality of evaluations for exemplar services or programs (Murad et al., 2017). Evaluations were assessed for (1) study limitations (including selection bias and incomplete outcome data), (2) adequacy of data (including concerns about sample size and analysis) and (3) indirectness/relevance (concerns about outcome measures). All records started at “high” certainty of evidence. The certainty was lowered (or downgraded) by one level for serious concerns or two levels for very serious concerns for each domain. This process resulted in an overall certainty rating of high (no downgrading), moderate (downgraded by one), low (downgraded by two) or very low (downgraded by three or more). Records that were peer-reviewed descriptive, observational and qualitative studies or mixed-method evaluation reports conducted by independent organisations began at “high”, while program descriptions, expert opinions, commentaries, annual reports or internal evaluations began at “low” and downgraded to “very low” as per the above criteria.

### Data analysis and presentation

The extracted data were summarised into simple descriptions and tabulated for each research question. For research question two, each exemplar service or program was mapped to an umbrella framework to assess its alignment with the guiding principles and service elements. For research questions three and four, exemplar services and programs were grouped by primary service type (justice and legal support; family violence prevention and/or support; primary health care with family and health promotion support; and broad community services) to assist with the organisation of the data. However, as no clear differences were observed across service types during the analysis, the findings were summarised collectively rather than reported separately by category. All analyses were undertaken using a strengths-based lens that centred on Indigenous values and perspectives throughout the analysis and reporting process.

## Results

### Search results

**Figure 1.**
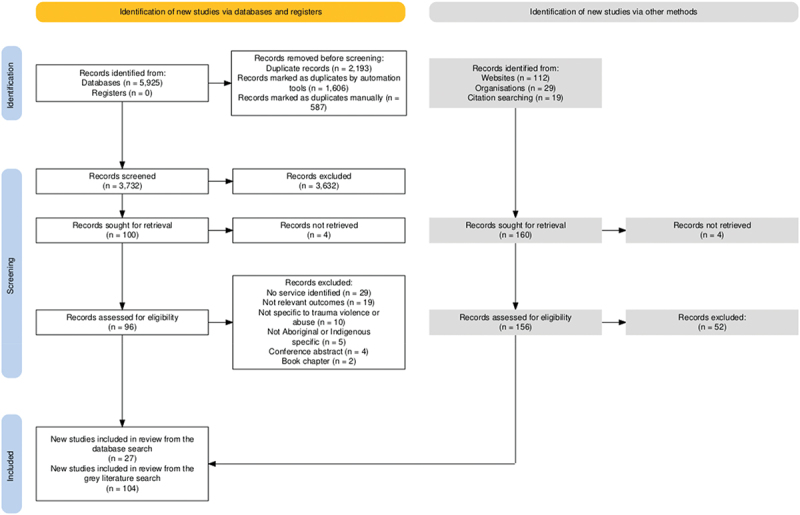
PRISMA flowchart.

Figure 1 displays the PRISMA flowchart of search results. Electronic searches yielded 5,925 records, of which 3,732 were screened based on title and abstract and 96 assessed for eligibility through a full-text review. Twenty-seven studies were deemed eligible for inclusion. Additional 156 records were identified through the grey literature search, of which 104 were deemed eligible for inclusion. In total, 131 records were included in the review. This includes 80 services and/or programs, of which 15 were rated as high relevance and quality, and included as exemplar services or programs. A detailed summary of each exemplar service or program is provided in Table A4 to Table A18 in Appendix 2. An overview of the remaining 65 services is provided in Table A19 in Appendix 2.

RQ1: ***What specific services have been developed for Aboriginal and Torres Strait Islander people affected by trauma, violence or abuse?***

#### Characteristics of all services and programs

The 80 identified services and/or programs were targeted at: general healing and social and emotional wellbeing (37%), supporting people with experiences of potentially traumatic events (23%), treatment of trauma symptoms (15%) or a mix (25%). Most services and/or programs were targeted at supporting Aboriginal and/or Torres Strait Islander adults and their families (68%), while others had a specific target group. Services and/or programs were identified in all states and territories in Australia, and most were still in operation (79%). Services and/or programs most commonly offered non-specific healing/social and emotional wellbeing support (74%), and counselling and therapeutic support (54%). See Table A20 in Appendix 3 for further details.

#### Characteristics of exemplar services or programs

Fifteen out of the 80 services and programs were rated as high relevance and quality and included as exemplar services or programs (Table 3). Services and programs operated across all states and territories except Tasmania and were delivered by Aboriginal Community Controlled Organisations (*n* = 9) or not-for-profit organisations that were Aboriginal-led, governed or founded (*n* = 6). Five services and/or programs were categorised as supporting people with experiences of potentially traumatic events, five as social and emotional wellbeing services, three as trauma-specific services and two as a mix. Services or programs were broadly categorised into the following service types: justice and legal support (*n* = 1); family violence prevention and/or support (*n* = 3); primary health care in addition to a range of family and health promotion supports (*n* = 3); or broad community services (*n* = 8). Some were available to all Aboriginal and/or Torres Strait Islander peoples, families or communities (*n* = 7), others were specifically for women or women and their children (*n* = 3), men (*n* = 2) or young people (*n* = 3). See Table A20 in Appendix 3 and Table A4 to Table A18 in Appendix 2 for further details on the characteristics of the exemplar services or programs.

**Table 3.** Exemplar services and programs.

Organisation	Relevant Programs
Victorian Aboriginal Child and Community Agency (VACCA) (Black et al., 2019, 2024)	1. Cultural Healing Programs
Dardi Munwurro (Deloitte Access Economics, 2021)	1. Men’s Healing and Behavioural Change 2. Bramunh Jaarn (Journeys Program) 3. Ngarra Jarranounith Place (residential program)
Deadly Connections Community Services (Booth, 2020; Deadly Connections, 2021)	1. Girra Girra Healing Place 2. Deadly Families 3. Deadly Young Warriors 4. Deadly Futures 5. Deadly Pathways 6. Deadly Brothers and Deadly Tiddas
Djirra (formerly Family Violence Prevention Legal Service – FVPLS) (Aboriginal Family Violence Prevention And Legal Service Victoria, 2014; Djirra, 2024)	1. Sisters Day Out 2. Dilly Bag 3. Dilly Bag: The Journey
Kornar Winmil Yunti (KWY) Aboriginal Corporation (Cahill et al., 2021)	1. Stronger, Safer Families Outreach Hubs 2. Aboriginal Women’s Safety Contact Service 3. Taikurtirna, Tirra-Apinthi 4. Intensive Family Support (Ana Wardli – Towards Home, Walking Together) 5. My Journey 6. Healing by Art program
Mpwelarre Health Aboriginal Corporation (Carey, 2013)	1. Social and Emotional Wellbeing Service
Murri School (Deloitte Access Economics, 2017)	1. Healing Program
Marninwarntikura Women’s Resource Centre (MWRC) (Marninwarntikura Women’s Resource Centre, 2021, 2022, 2023, 2024; Pearce, 2017)	1. Baya Gawaiy Buga Yani Jandu Yani U 2. Crisis Response (Family Violence Prevention Legal Unit) 3. Marulu 4. Marnin Studio
North Australian Aboriginal Justice Agency (NAAJA) (Prince, 2021)	1. Healing Program
Neami National (Chiera, 2021)	1. Wadamba Wilam
Ngaanyatjarra Pitjantjatjara Yankunytjatjara Women’s Council (NPYWC) (Togni, 2019)	1. Youth Services 2. Child & Family Wellbeing Service 3. Tjungu Aged & Disability Service 4. Domestic & Family Violence Service (including Uti Kulin Tjaku Watiku Project) 5. Tjanpi Desert Weavers Social Enterprise 6. Ngangkari Traditional Healing
Ngaoara (Ngaoara Limited, 2019)	1. Trauma Assessment, Referral and Rehabilitation Outreach Teams (TARROT)
Healing Foundation (The Healing Foundation, 2015)	1. Our Men Our Healing Project
Red Dust Healing (Dust, 2018)	1. Red Dust Healing Program
Waminda – South Coast Women’s Health and Welfare Aboriginal Corporation (Armstrong, 2019; SNAICC, 2021; Waminda, 2022)	1. Nabu Aboriginal Family Preservation and Restoration Program 2. Waminda Counselling Services 3. Baalang Healing 4. Case Management Services 5. Strong Yawa

RQ2: ***What are the guiding principles or values underpinning these services?***

Table 4 illustrates the alignment of each exemplar service or program with the guiding principles and service elements of the consensus umbrella framework (see Table 1). Eight of the 15 services mentioned the importance of acknowledging history and the impacts of colonisation (which was implied in additional four services); 12 were trauma-aware and healing-informed; and 12 explicitly prioritised self-determination and empowerment (details of the services and programs included in each category are provided in Table 4). All 15 services were culturally centred; 13 services explicitly included culture as one of their guiding principles, while two services implied cultural grounding, safety and connection within the core components of service delivery. All but two services were community-led; 13 emphasised safety, collaborative and holistic approaches; and 13 provided flexible, tailored responses for individuals and communities. There was greater uncertainty on whether services integrated strengths-based elements into their services or emphasised the incorporation of evaluation frameworks for monitoring program effectiveness and sustainability. However, as 13 out of the 15 services had evaluation evidence available, they may have evaluation frameworks in place that were not explicitly reported.

**Table 4.** Overarching guiding principles, evidence-informed service elements and core components of exemplar services or programs, categorised by primary service types, with tallies for each category.

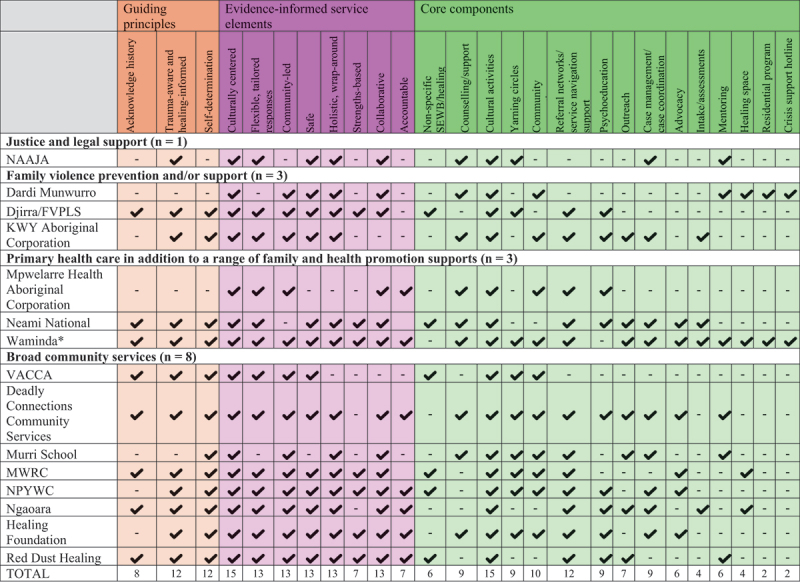

*Notes*. * = Services have underpinning frameworks but do not have available evaluation evidence; - = elements were not clearly described. Family Violence Prevention Legal Service (FVPLS), Kunar Winmil Yunti (KWY), Trauma Assessment, Referral & Rehabilitation Outreach Teams (TARROT), Ngaanyatjarra Pitjantjatjara Yankunytjatjara Women’s Council (NPYWC).

RQ3: ***What are the core components of these services?***

**Figure 2.**
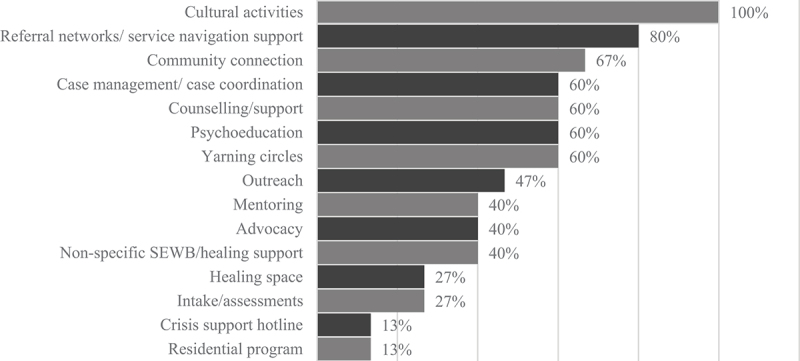
Proportion of exemplar services or programs offering service components (*n* = 15).

A summary of the core components of each exemplar service or program is provided in Figure 2 and Table 4, which is organised by primary service type.

Overall, all 15 services integrated *culturally grounded activities* into their service delivery models. These activities served both therapeutic and cultural identity strengthening purposes and included yarning, storytelling and cultural activities, such as ceremonies, art, possum skin cloak making and camps or time on Country.

Nine services outlined *additional therapeutic interventions* including individual and/or group counselling; behaviour change workshops; family, narrative, art and play therapies; and traditional healing methods. Additionally, psychoeducation was offered by nine services and 12 services offered referral networks and/or support to navigate service systems.

Nine provided *additional wrap-around support* via case management and/or care coordination. Two services demonstrated this support implicitly; one through the provision of supports across a range of domains, including employment, training, relationships and parenting. The second service demonstrated this through collaboration between SEWB service staff and allied health and health service teams.

*Community and/or family connection* was detailed by 10 services as a fundamental component. These services offered a range of activities to strengthen this connection, including tracing family history and cultural tours, providing safe spaces to facilitate reconnection with culture and community, time spent on Country and time with Elders.

*Outreach activities* were core components in seven services, and advocacy was the core component in six service delivery models, which included parliamentary submissions, legal support and promotion of local and systemic change through actions, such as cultural immersion workshops and meetings with services. Mentoring was offered by six services.

The *size and composition of staffing* varied across the 15 services. Most employed multidisciplinary teams, including psychology, medical and allied health, youth and family support and engagement, and social work. These services provided a range of specialised counselling, including Aboriginal healing, social and emotional wellbeing, youth, child trauma and men’s and women’s family violence. Five programs were delivered by facilitators; two programs were delivered by Aboriginal facilitators. One service engaged facilitators and community Elders to deliver a behaviour change program. Six services explicitly noted that non-Aboriginal staff completed cultural training and mentorships or had extensive prior experience working with and within Aboriginal and Torres Strait Islander communities. One service prioritised the employment of both Aboriginal and non-Aboriginal staff with lived experience of healing and systems involvement.

RQ4: ***Are there any key success factors underpinning delivery (e.g., effectiveness, acceptability and feasibility)?***

A detailed summary of the evaluation indicators of each exemplar service or program is provided in Table A4 to Table A18 in Appendix 2.

#### Quality assessment of evaluation reports

Evaluations from three services were assessed as high certainty of evidence, seven as moderate, two as low and three as very low (see Table A21 in Appendix 3). Most studies were downgraded due to study limitations and/or data accuracy, including limited information provided about how data were collected, analysed or interpreted.

#### Effectiveness

Effectiveness outcomes were reported for all but one service. Most services reported trends of improvement as self-reported social and emotional wellbeing, including increased connection to culture, community, spirit and Country, improved relationships with family and increased feelings of safety, improvements in confidence, feelings of positivity and empowerment, improved life satisfaction, improved ability to express emotions and process grief and loss, greater self-awareness, ability to make beneficial choices and strengthened conflict resolution skills (Aboriginal Family Violence Prevention And Legal Service Victoria, 2014; Black et al., 2019; Booth, 2020; Chiera, 2021; Deadly Connections, 2021; Deloitte Access Economics, 2017, 2021; Dust, 2018; The Healing Foundation, 2015; Marninwarntikura Women’s Resource Centre, 2021, 2022; Ngaoara Limited, 2019; Prince, 2021; Togni, 2019).

One service measured wellbeing through the Strengths and Difficulties Questionnaire and reported reductions in “borderline” and “abnormal” scores (Deloitte Access Economics, 2017). A number of evaluations did not specify how wellbeing was measured, however their delivery models were centred on holistic understandings of wellbeing. Many services reported improvements in individual principles of the SEWB model, such as cultural connectedness (Black et al., 2019; Booth, 2020; Deadly Connections, 2021; Deloitte Access Economics, 2017, 2021; Dust, 2018; The Healing Foundation, 2015; Prince, 2021), and improvements in kinship, family and community relationships (Booth, 2020; Carey, 2013; Deadly Connections, 2021; Deloitte Access Economics, 2021; Dust, 2018; The Healing Foundation, 2015; Prince, 2021). Four services reported improvements in care and service coordination through increased collaboration (Aboriginal Family Violence Prevention And Legal Service Victoria, 2014; Deadly Connections, 2021; Ngaoara Limited, 2019; Prince, 2021). Additional outcomes reported included reductions in justice system involvement (Deloitte Access Economics, 2017, 2021; The Healing Foundation, 2015), improvement in educational engagement/attendance (Deloitte Access Economics, 2017, 2021; Ngaoara Limited, 2019), reductions in child protection notifications and removals (Deadly Connections, 2021; Deloitte Access Economics, 2017; SNAICC, 2021; Waminda, 2022) and rates of family violence (Deloitte Access Economics, 2021; The Healing Foundation, 2015) and alcohol and drug use (Chiera, 2021).

Two service evaluations reported positive returns on investment. One service delivered AUD$8.85 worth of return benefits for every dollar invested, in the form of cost savings in the child protection and justice systems and in increased educational attainment, which results in reduced dependence on financial support systems (Deloitte Access Economics, 2017). Another service saw 50–190% return on each dollar invested in their behaviour change programs (Deloitte Access Economics, 2021).

#### Acceptability

Indicators of acceptability similarly varied across the evaluated services. These included rates of engagement (Aboriginal Family Violence Prevention And Legal Service Victoria, 2014; Deadly Connections, 2021; Deloitte Access Economics, 2017; Dust, 2018; The Healing Foundation, 2015; Ngaoara Limited, 2019; Togni, 2019; Waminda, 2022), satisfaction ratings (Dust, 2018; Prince, 2021), positive participant feedback (Aboriginal Family Violence Prevention And Legal Service Victoria, 2014; Black et al., 2019; Carey, 2013; Deadly Connections, 2021; Deloitte Access Economics, 2017) and community and family appraisals (Deadly Connections, 2021; Dust, 2018; Ngaoara Limited, 2019). Acceptability indicators were not reported for three services (Cahill et al., 2021; Deloitte Access Economics, 2021; Marninwarntikura Women’s Resource Centre, 2021).

#### Feasibility

Feasibility was assessed through enablers and barriers to service implementation. Frequently reported enablers included the incorporation of cultural elements, cultural safety and connection (Black et al., 2019; Booth, 2020; Chiera, 2021; Deadly Connections, 2021; Dust, 2018; The Healing Foundation, 2015; Togni, 2019; Waminda, 2022) and employment of Aboriginal staff, local staff and/or staff with lived-experience (Aboriginal Family Violence Prevention And Legal Service Victoria, 2014; Cahill et al., 2021; Deadly Connections, 2021; Deloitte Access Economics, 2017; Marninwarntikura Women’s Resource Centre, 2021; Ngaoara Limited, 2019). Additional enablers included adaptability to local needs, accessibility and flexibility (Chiera, 2021; Deadly Connections, 2021; Dust, 2018; The Healing Foundation, 2015; Waminda, 2022), partnerships and collaboration with other teams and services (Chiera, 2021; Ngaoara Limited, 2019; Prince, 2021), commitment to clients and belief in the program (Cahill et al., 2021; Deadly Connections, 2021; Ngaoara Limited, 2019), skills and capacity development (Marninwarntikura Women’s Resource Centre, 2021; Ngaoara Limited, 2019; Prince, 2021), trust (Aboriginal Family Violence Prevention And Legal Service Victoria, 2014; Carey, 2013; Deloitte Access Economics, 2017; Dust, 2018), teamwork (Carey, 2013; Deloitte Access Economics, 2017) and good staff-to-case ratio (Chiera, 2021).

Barriers related to inadequate resourcing were a recurrent theme across the evaluations, including funding constraints (Carey, 2013; Deadly Connections, 2021; The Healing Foundation, 2015; Marninwarntikura Women’s Resource Centre, 2021; Waminda, 2022); staff and workforce challenges, such as staff shortages, heavy workloads and workforce casualisation (Cahill et al., 2021; Carey, 2013; Deadly Connections, 2021; The Healing Foundation, 2015; Marninwarntikura Women’s Resource Centre, 2021; Pearce, 2017); inability to meet high demands (Aboriginal Family Violence Prevention And Legal Service Victoria, 2014; Dust, 2018; Ngaoara Limited, 2019; SNAICC, 2021); lack of physical space (The Healing Foundation, 2015; Prince, 2021) and difficulties with data collection and management (Booth, 2020; Cahill et al., 2021; Deadly Connections, 2021), or with referral and case management systems. Communication difficulties with clients were highlighted by two services (Cahill et al., 2021; Carey, 2013), one of these also highlighted issues with collaboration with other services and lack of clear decision-making pathways as additional barriers affecting the feasibility of the service (Carey, 2013). One service highlighted the lack of culturally appropriate child risk assessment measures as a barrier (Cahill et al., 2021), another noted the lack of available services in remote areas (Dust, 2018), and one pointed to external policy decisions as hindering effective service delivery (Prince, 2021). Barriers were not reported for five services (Black et al., 2019; Chiera, 2021; Deloitte Access Economics, 2017, 2021; Prince, 2021; Togni, 2019).

## Discussion

Our findings underscore the importance of community-controlled, culturally grounded and self-determined approaches to healing services, incorporating holistic, wrap-around support to strengthen cultural, community and family connections. Despite many services and programs, there are limited published program evaluations.

The guiding principles and service elements observed in exemplar services or programs closely align with broader policy and research recommendations, reflecting widely recognised standards of acceptable and appropriate service delivery. For example, the National Aboriginal and Torres Strait Islander Health Plan (NATSIHP) 2021–2031 embeds a *healing as recovery* model that recognises the impacts of colonisation, racism and intergenerational trauma, while also drawing on cultural strengths to promote holistic wellbeing. Victorian frameworks echo this approach: Strategic Direction Four of the Victorian Health and Wellbeing Strategic Plan 2023–2027 (Department of Health, 2025) and the Victorian Aboriginal Health and Wellbeing Partnership Agreement (2023–2033) (Victorian Aboriginal Community Controlled Health Organisation, 2022) and Action Plan (2023–2025) (Victorian Aboriginal Community Controlled Health Organisation, 2023), all emphasise community-led, culturally grounded, safe, strengths-based and self-determined approaches to health and wellbeing. Comparable themes are evident in other reviews on Aboriginal and Torres Strait Islander healing and SEWB services, which highlight the centrality of culture, empowerment and healing-informed care (Gupta et al., 2020; Horak & Thompson, 2025; McKendrick et al., 2013; Murrup-Stewart et al., 2025; Roy et al., 2015; Wise et al., 2024).

Parallels also emerge in the international Indigenous health literature. Yu et al. (2020), for instance, reviewed Canadian healing programs and found recurring principles such as honouring culture and tradition, adopting strengths-based and empowering approaches, and applying the Medicine Wheel framework, which integrates physical, emotional, mental and spiritual wellbeing. Their study emphasized engagement and ownership, local tailoring, collaboration, family and community centredness, and trauma-informed practices. Models of healing services and trauma-informed care for Māori in Aotearoa, New Zealand (Dobbs & Eruera, 2014; Pihama et al., 2017; Wilson et al., 2021; Wirihana & Smith, 2019) and Indigenous peoples in the United States (King & Guillory, 2015; Oré et al., 2025) likewise highlight these principles, underscoring their global relevance across Indigenous contexts.

Finally, the principles identified in this review resonate with Western trauma-informed care models. The Substance Abuse and Mental Health Services Administration’s trauma-informed care principles, for example, include safety, trust, collaboration, empowerment and cultural responsiveness (Center for Substance Abuse Treatment (US), 2014). This convergence suggests that while services for Aboriginal and Torres Strait Islander peoples are distinct in their grounding in culture, history and self-determination, they are also consistent with broader understandings of trauma-informed practice. Importantly, embedding healing-oriented principles shifts trauma discourse away from deficit-based framings of, “What is wrong with you?”, towards perspectives that acknowledge lived experiences, including “What has happened to you?” and “How have you survived?” (Grossman et al., 2021; Johnstone et al., 2018), and strengths, “What is right with you?” (Ginwright, 2018). Such an approach not only recognises the enduring impacts of colonisation and systemic injustice but also affirms cultural strength, connection and empowerment as foundations for healing (Healing Foundation, 2020).

While the core components of exemplar services or programs varied, cultural practices emerged as the most prominent feature, representing tangible applications of the guiding principles and service elements. This finding aligns with broader evidence emphasising the centrality of culturally grounded practices in effective service delivery (Horak & Thompson, 2025; Murrup-Stewart et al., 2025). The prominence of these practices reflects the growing recognition of the importance of cultural determinants of health, defined as factors that help support the health and wellbeing of Aboriginal and Torres Strait Islander peoples (Brady et al., 2024; Fields et al., 2024; Kelly et al., 2024; Lovett et al., 2020; Lowitja Institute, 2020). These determinants include connection to Country; family, kinship, and community; Indigenous beliefs and knowledge; cultural expression and continuity; language; and self-determination and leadership (Kelly et al., 2024; Salmon et al., 2018). Within the SEWB model, cultural determinants are positioned as equally influential as social, political and historical factors in shaping health and wellbeing (Gee et al., 2014). Connection to culture and country are also key themes in national strategies to improve SEWB among Aboriginal and Torres Strait Islander communities, including the NATSIHP 2021–2031 (Department of Health, 2021).

Strengths-based approaches that draw on cultural determinants have been associated with improved health and wellbeing outcomes (Dudgeon et al., 2021; Murrup-Stewart et al., 2025; Victorian Aboriginal Community Controlled Health Organisation, 2022). This is important given that low self-esteem is a key diagnostic feature of complex trauma (Cloitre et al., 2018), making strengths-based strategies a key therapeutic pathway. In the Australian context, examples include suicide prevention initiatives in the Tiwi Islands, which emphasised reconnection to self, culture, ceremony, language, skin groups and Country as contributing to reductions in suicide rates from the early 2000s (Prince, 2018). Other initiatives have focused on language revitalisation (Sivak et al., 2019), incorporating the sounds of Country into prison programs (Marchetti et al., 2022) and facilitating on-Country camps (Brady et al., 2024; Yashadhana et al., 2024), further reinforcing the relationship between cultural continuity and wellbeing. Nationally, longitudinal research is underway examining the role of cultural determinants in promoting Aboriginal and Torres Strait Islander health and wellbeing (Jones et al., 2018; Lovett et al., 2020). Internationally, similar patterns have been observed: in Aotearoa, New Zealand, the Māori renaissance led to sustained health gains for Indigenous communities (Kunitz, 1994), while in Canada and the United States, programs fostering cultural continuity have demonstrably enhanced the health and wellbeing of Indigenous peoples (Auger, 2016; Chandler & Lalonde, 1998; Sowerwine et al., 2023). The integration of cultural components in these examples, alongside the exemplar services or programs, illustrates how cultural determinants operate as protective factors, embedding holistic approaches that enhance SEWB through sustained cultural connections (Bourke et al., 2022; Gee et al., 2014; Kelly et al., 2024; Verbunt et al., 2021).

Services were generally regarded as acceptable, as evidenced by high levels of engagement and positive participant feedback. This is important as acceptability forms a foundation for the effectiveness and impact of population-based services (Sekhon et al., 2017). Conversely, the absence of acceptable and culturally safe options has been identified as a key barrier to accessing mainstream health, trauma and healing services (Goetz et al., 2023; Herring et al., 2013; Nolan-Isles et al., 2021; Rodaughan et al., 2024). Services were also reported to be feasible; however, inadequate resourcing was identified as a barrier to service implementation, a finding consistent with previous studies (Dossetor et al., 2023). Continuous, non-project-based funding is essential to sustain program development and evaluation processes, enabling services to demonstrate impact, support further investment and build workforce and health service capacity. This ensures that programs remain viable beyond the lifespan of project or research grants and continue to evolve in ways that are accountable to, and led by, communities (Kushnier et al., 2023).

Evaluations are often important for improving funding sustainability; however, only a minority of services and/or programs had evaluation reports suitable for inclusion in this review. This limitation has been noted in other publications on Aboriginal and Torres Strait Islander health services (Hudson, 2016). Even among the included exemplar service or program evaluations, differences existed in how success or effectiveness was defined and measured. For instance, some services defined effectiveness through a SEWB lens, focusing on indicators, such as cultural connection, emotional wellbeing and community ties, while others reported reductions in family violence, rates of homelessness, child protection notifications or substance use. This diversity highlights that there is no single definition of success and underscores the need to account for contextual and cultural differences when designing evaluation strategies. This ensures that success factors are unique to the service, meaningful to communities and that the value of the evaluation outcomes are recognised by potential funders (Maddox et al., 2021).

However, measuring success within Indigenous health services and SEWB programs remains challenging (Cargo et al., 2019; Finlay et al., 2021; Kelaher et al., 2018; McKendrick et al., 2013). A key issue is reconciling Western evaluation frameworks with Aboriginal and Torres Strait Islander worldviews (Roy et al., 2015; Stelkia et al., 2023; Venugopal et al., 2021). While Western paradigms often emphasise quantitative measures and standardised reporting, Indigenous approaches prioritise holistic, qualitative understandings of healing and success (Bulloch et al., 2019). This includes oral histories, storytelling and narrative-based methods, which better reflect the ongoing and personal nature of the healing journey (Kushnier et al., 2023). These journeys are culturally grounded and interconnected with identity, community and Country, making them difficult to capture through conventional metrics (Arnott et al., 2010; Roy et al., 2015). Defining healing through numbers alone risks overlooking the intangible, but vital elements valued by communities, such as connection to culture, language and belonging (Arnott et al., 2010; Bourke et al., 2022; Murrup-Stewart et al., 2021; Roy et al., 2015). Recognition of these needs is reflected in the increasing number of Aboriginal and Torres Strait Islander evaluation frameworks being developed (e.g., Children’s Ground, n.d.; Inside Policy, 2023; Kelaher et al., 2018; Kildea et al., 2016; Productivity Commission, 2020; QATSICPP, n.d.; SNAICC, n.d.).

### Implications for policy, practice and research

The findings of this review can be used to (re)design services and/or programs aimed at improving healing, health and wellbeing outcomes for Aboriginal and Torres Strait Islander people affected by trauma, violence and abuse. To be effective, this work must occur in close consultation with local communities to ensure that services are culturally responsive and meet local community-identified needs and differences. Investment is also required to support the inclusion of cultural components within services, not as supplementary to mainstream approaches, but as central to effective healing and wellbeing services.

There is also a need to strengthen the Aboriginal and Torres Strait Islander health evidence base through dedicated research and evaluation. A key barrier to achieving this, however, is the disconnect between communities, the community-controlled sector, governments and funding bodies in how evidence is generated, interpreted and applied (Kushnier et al., 2023). Addressing this disconnect requires the decolonisation of program development and evaluation processes. Indigenous knowledge systems must lead and shape the design and implementation of healing services and evaluation processes, ensuring approaches are strengths-based, community-driven and reflective of Indigenous ways of knowing, being and doing (Productivity Commission, 2020). It will also ensure that the needs of individuals affected by trauma, violence and abuse are prioritised. Respect for Indigenous data sovereignty is important to this shift, as it affirms communities’ rights to govern the collection, ownership and use of data about their programs and people. Upholding these principles allows evaluation evidence to be generated, interpreted and applied in self-determined ways that both strengthen accountability and promote empowerment (Lowitja Institute, 2024).

### Limitations

It is important to acknowledge several limitations to the review. It remains unclear whether the additional 64 services and/or programs identified in the review have been evaluated. We contacted organisations when we suspected that evaluations had been conducted to mitigate this limitation. In addition, the specific focus on trauma-related services in our search was necessary given the scope of this review; however, this may have resulted in the exclusion of adjacent services (such as alcohol and drug services). Finally, the quality of some evaluations of included exemplar services or programs was judged to be low or very low. Some evaluations relied on internal reports, some had incomplete datasets, others had low response rates or did not clearly describe analytical methods (including how data were collected, analysed or interpreted). As a result, the overall strength and comparability of the evaluation evidence was limited.

## Conclusions

This scoping review provides an overview on healing services for Aboriginal and Torres Strait Islander people affected by trauma, violence or abuse. It is evident from the broader literature that acknowledging the historical context and enduring impacts of colonisation is of primary importance for any service designed for Aboriginal and Torres Strait Islander people. Use of a trauma-aware and healing-informed approach that prioritises the self-determination and empowerment of individuals and communities is also necessary. Additionally, services should be firmly rooted in the local cultural context, led by the community with safety and have a strength-based perspective in mind. There should be an emphasis on collaboration and offering flexible, personalised wrap-around responses that encompass both preventative and restorative measures, while maintaining a holistic outlook. Services should incorporate culturally appropriate evaluation frameworks, which not only ensure accountability but also promote transparency and program sustainability.

These guiding principles, service elements and core components can inform service design and delivery, offering a framework that can be strengthened through ongoing collaboration with local communities, the community-controlled sector, governments and funding bodies. More evaluations of existing services are needed, and these must be funded, designed, conducted and reported in ways that stay grounded in the community and true to Aboriginal and Torres Strait Islander ways of knowing, being and doing.

## Supplementary Material

Appendices

## Data Availability

The data supporting this scoping review are derived from publicly available sources, including peer-reviewed journal articles, grey literature and other relevant documents identified through systematic database searches and non-systematic grey literature and snowball searches. The datasets generated and analysed during this study are included in the manuscript and its supplementary materials, where applicable. Additional details regarding the search strategy, data extraction and synthesis can be obtained from the corresponding author upon reasonable request.
