# Supplementary material for: Healing and trauma services for Aboriginal and Torres Strait Islander people: a scoping review
Source: Aust J Psychol. 2026 Jun 11;78(1):2678850. doi: 10.1080/00049530.2026.2678850 (PMC13262101; doi:10.1080/00049530.2026.2678850)
Supplement: Appendices [file RAUP_A_2678850_SM3294.docx]

# Appendices

## Appendix 1: Supplementary methods

### **CONSIDER Statement Checklist**

| Item | | Page |
| --- | --- | --- |
| Governance | |  |
| 1. | Describe partnership agreements between the research institution and Indigenous-governing organization for the research, (e.g., Informal agreements through to MOU (Memorandum of Understanding) or MOA (Memorandum of Agreement)). | 4 |
| 2. | Describe accountability and review mechanisms within the partnership agreement that addresses harm minimization. | N/A |
| 3. | Specify how the research partnership agreement includes protection of Indigenous intellectual property and knowledge arising from the research, including financial and intellectual benefits generated (e.g., development of traditional medicines for commercial purposes or supporting the Indigenous community to develop commercialization proposals generated from the research). | N/A |
| Prioritization | |  |
| 4. | Explain how the research aims emerged from priorities identified by either Indigenous stakeholders, governing bodies, funders, non-government organization(s), stakeholders, consumers, and empirical evidence | 4 |
| Relationships (Indigenous stakeholders/participants and Research team) | |  |
| 5. | Specify measures that adhere and honour Indigenous ethical guidelines, processes, and approvals for all relevant Indigenous stakeholders, recognizing that multiple Indigenous partners may be involved, e.g., Indigenous ethics committee approval, regional/national ethics approval processes. | N/A |
| 6. | Report how Indigenous stakeholders were involved in the research processes (i.e., research design, funding, implementation, analysis, dissemination/recruitment). | 4 |
| 7. | Describe the expertise of the research team in Indigenous health and research. | 4 |
| Methodologies | |  |
| 8. | Describe the methodological approach of the research including a rationale of methods used and implication for Indigenous stakeholders, e.g., privacy and confidentiality (individual and collective) | 4-7 |
| 9. | Describe how the research methodology incorporated consideration of the physical, social, economic and cultural environment of the participants and prospective participants. (e.g., impacts of colonization, racism, and social justice). As well as Indigenous worldviews. | 3  22 |
| Participation | |  |
| 10. | Specify how individual and collective consent was sought to conduct future analysis on collected samples and data (e.g., additional secondary analyses; third-parties accessing samples (genetic, tissue, blood) for further analyses). | N/A |
| 11. | Described how the resource demands (current and future) placed on Indigenous participants and communities involved in the research were identified and agreed upon including any resourcing for participation, knowledge, and expertise | N/A |
| 12. | Specify how biological tissue and other samples including data were stored, explaining the processes of removal from traditional lands, if done, and of disposal. | N/A |
| Capacity | |  |
| 13. | Explain how the research supported the development and maintenance of Indigenous research capacity (e.g., specific funding of Indigenous researchers). | N/A |
| 14. | Discuss how the research team undertook professional development opportunities to develop the capacity to partner with Indigenous stakeholders? | N/A |
| Analysis and interpretation | |  |
| 15. | Specify how the research analysis and reporting supported critical inquiry and a strength-based approach that was inclusive of Indigenous values. | 4 |
| Dissemination | |  |
| 16. | Describe the dissemination of the research findings to relevant Indigenous governing bodies and peoples. | This paper was initially published as an internal report for VACCA, with findings presented to the organisation. Results were also shared through an oral presentation and poster presentation at the Lowitja Institute’s 4th International Indigenous Health and Wellbeing Conference in June 2025. |
| 17. | Discuss the process for knowledge translation and implementation to support Indigenous advancement (e.g., research capacity, policy, investment). | N/A |

### **Table A1.** Eligibility criteria

| **Inclusion Criteria** | |
| --- | --- |
| ***Population*** | - Aboriginal and Torres Strait Islander peoples from Australia - Any age, gender or health status |
| ***Concept*** | Trauma-specific services or programs designed to support Indigenous people with a lived experience of trauma  Trauma, violence or abuse-specific services or programs defined as:   - Services with a focus on delivery - Provides a range of care, support or intervention options for people experiencing trauma to meet their needs - Staff can deliver multiple interventions within the service   A service was defined as an organisational structure delivering trauma-related care or support. A program was defined as a set of activities or interventions delivered within a service or organisation. |
| ***Context*** | - Australia - Various settings: health care, community, school |
| ***Other*** | **Outcomes**: Service characteristics of interest:   - Theoretical, epistemological, conceptual basis of the service - Guiding principles or values - Main components of the service (e.g., the process of healing, Models of care) - Human resources (e.g., setting/who delivers the intervention and qualifications required) - Key success factors (e.g., acceptability, feasibility, cost, effectiveness, barriers/enablers of care) |
|  | **Sources**   - Peer-reviewed literature, including primary research articles or papers (quantitative, qualitative and mixed methods designs); review articles (including systematic and scoping reviews); text, opinion, commentary and discussion papers. - Grey literature, including academic, governmental and organisational reports, websites and opinions pieces - Papers published in English. |
| **Exclusion Criteria** | |
| ***Population*** | Non-Indigenous peoples |
| ***Concept*** | Individual-level trauma-focused interventions, including single, multiple or tailored interventions |
| ***Context*** | NA |
| ***Other*** | - Theses/dissertations - Papers not published in English |

### Search strategy

**Medline: 527 results on July 19, 2023, updated July 2, 2025**

| 1 | exp Indigenous Peoples/ |
| --- | --- |
| 2 | (aborigin* or "first nations" or "first nation" or indigenous or first people*).mp. |
| 3 | ("Torres Strait Islander*" or Maori* or "American Indian*" or "Alask* Nativ*" or "Nativ* Alask*" or "Nativ* Hawaiian*" or "Hawaii* Nativ*" or "Nativ* Americ*" or "Americ* Nativ*" or "Americ* Samoa*" or "Samoa* Americ*" or Eskimo* or Inuit* or Aleut* or Metis).mp. |
| 4 | Stress Disorder*.mp. |
| 5 | ("PTSD" or "posttrauma*" or "post-trauma*" or "post trauma*" or "stress disorder*").mp. |
| 6 | ("out of home care").mp. |
| 7 | (((History or histories or historic* or survivor* or continuity or cycle* or discontinuity or disclos* or recover* or unresolved or past or cultur* or coloni* or generation* or intergeneration* or inter-generation*) adj2 (abus* or adversity or maltreat* or neglect* or posttrauma* or post-trauma* or stress or trauma* or violen*)) not ("physical trauma" or "head trauma" or "traumatic brain injury")).mp. |
| 8 | Health Services Indigenous/ |
| 9 | (Service* or approach* or strateg* or program* or framework* or practice* or tool* or casework*).mp. |
| 10 | ((trauma* or cultur*) adj2 inform*).mp. |
| 11 | (healing or wellbeing or "well being" or "strength based" or holistic or integrat*).mp. |
| 12 | 1 or 2 or 3 |
| 13 | 4 or 5 or 6 |
| 14 | 7 or 8 or 9 or 10 |
| 15 | 12 and 13 and 14 |
| 16 | limit 15 to (english language and yr="2012 -Current") |

**PsycINFO: 717 results on July 19, 2023, updated July 2, 2025**

| 1 | exp Indigenous Populations/ |
| --- | --- |
| 2 | (aborigin* or "first nations" or "first nation" or indigenous or first people*).mp. |
| 3 | ("Torres Strait Islander*" or Maori* or "American Indian*" or "Alask* Nativ*" or "Nativ* Alask*" or "Nativ* Hawaiian*" or "Hawaii* Nativ*" or "Nativ* Americ*" or "Americ* Nativ*" or "Americ* Samoa*" or "Samoa* Americ*" or Eskimo* or Inuit* or Aleut* or Metis).mp. |
| 4 | Stress Disorder*.mp. |
| 5 | ("PTSD" or "posttrauma*" or "post-trauma*" or "post trauma*" or "stress disorder*").mp. |
| 6 | ("out of home care").mp. |
| 7 | (((History or histories or historic* or survivor* or continuity or cycle* or discontinuity or disclos* or recover* or unresolved or past or cultur* or coloni* or generation* or intergeneration* or inter-generation*) adj2 (abus* or adversity or maltreat* or neglect* or posttrauma* or post-trauma* or stress or trauma* or violen*)) not ("physical trauma" or "head trauma" or "traumatic brain injury")).mp. |
| 8 | Health Care Services/ |
| 9 | (Service* or approach* or strateg* or program* or framework* or practice* or tool* or casework*).mp. |
| 10 | ((trauma* or cultur*) adj2 inform*).mp. |
| 11 | (healing or wellbeing or "well being" or "strength based" or holistic or integrat*).mp. |
| 12 | 1 or 2 or 3 |
| 13 | 4 or 5 or 6 |
| 14 | 7 or 8 or 9 or 10 |
| 15 | 12 and 13 and 14 |
| 16 | limit 15 to (english language and yr="2012 -Current") |

**Embase: 721** **results on July 19, 2023, updated July 2, 2025**

| 1 | exp Indigenous People/ or Indigenous Australian/ |
| --- | --- |
| 2 | (aborigin* or "first nations" or "first nation" or indigenous or first people*).mp. |
| 3 | ("Torres Strait Islander*" or Maori* or "American Indian*" or "Alask* Nativ*" or "Nativ* Alask*" or "Nativ* Hawaiian*" or "Hawaii* Nativ*" or "Nativ* Americ*" or "Americ* Nativ*" or "Americ* Samoa*" or "Samoa* Americ*" or Eskimo* or Inuit* or Aleut* or Metis).mp. |
| 4 | Stress Disorder*.mp. |
| 5 | ("PTSD" or "posttrauma*" or "post-trauma*" or "post trauma*" or "stress disorder*").mp. |
| 6 | ("out of home care").mp. |
| 7 | (((History or histories or historic* or survivor* or continuity or cycle* or discontinuity or disclos* or recover* or unresolved or past or cultur* or coloni* or generation* or intergeneration* or inter-generation*) adj2 (abus* or adversity or maltreat* or neglect* or posttrauma* or post-trauma* or stress or trauma* or violen*)) not ("physical trauma" or "head trauma" or "traumatic brain injury")).mp. |
| 8 | Health Service/ |
| 9 | (Service* or approach* or strateg* or program* or framework* or practice* or tool* or casework*).mp. |
| 10 | ((trauma* or cultur*) adj2 inform*).mp. |
| 11 | (healing or wellbeing or "well being" or "strength based" or holistic or integrat*).mp. |
| 12 | 1 or 2 or 3 |
| 13 | 4 or 5 or 6 |
| 14 | 7 or 8 or 9 or 10 |
| 15 | 12 and 13 and 14 |
| 16 | limit 15 to (english language and yr="2012 -Current") |

**Web of Science**: **1445 results on July 19, 2023, updated July 2, 2025**

| 1 | TS=(aborigin* or "first nations" or "first nation" or indigenous or "first people"*) |
| --- | --- |
| 2 | TS=("Torres Strait Islander*" or Maori* or "American Indian*" or "Alask* Nativ*" or "Nativ* Alask*" or "Nativ* Hawaiian*" or "Hawaii* Nativ*" or "Nativ* Americ*" or "Americ* Nativ*" or "Americ* Samoa*" or "Samoa* Americ*" or Eskimo* or Inuit* or Aleut* or Metis) |
| 3 | TS=("PTSD" or "posttrauma*" or "post-trauma*" or "post trauma*" or "stress disorder*" |
| 4 | TS=("out of home care") |
| 5 | TS=((History or histories or historic* or survivor* or continuity or cycle* or discontinuity or disclos* or recover* or unresolved or past or cultur* or coloni* or generation* or intergeneration* or inter-generation*) NEAR/2 (abus* or adversity or maltreat* or neglect* or posttrauma* or post-trauma* or stress or trauma* or violen*)) |
| 6 | (TS=((History or histories or historic* or survivor* or continuity or cycle* or discontinuity or disclos* or recover* or unresolved or past or cultur* or coloni* or generation* or intergeneration* or inter-generation*) NEAR/2 (abus* or adversity or maltreat* or neglect* or posttrauma* or post-trauma* or stress or trauma* or violen*))) NOT TS=("physical trauma" or "head trauma" or "traumatic brain injury") |
| 7 | TS=(Service* or approach* or strateg* or program* or framework* or practice* or tool* or casework*) |
| 8 | TS=((trauma* or cultur*) NEAR/2 inform*) |
| 9 | TS=(healing or wellbeing or "well being" or "strength based" or holistic or integrat*) |
| 10 | #1 OR #2 |
| 11 | #3 OR #4 OR #6 |
| 12 | #7 OR #8 OR #9 |
| 13 | #10 AND #11 AND #12 |
| 14 | #13 Timespan: 2012-01-01 to 2023-12-31 |

**CINAHL: 440 results on July 19, 2023, updated July 2, 2025**

| 1 | (MH "Indigenous Peoples") |
| --- | --- |
| 2 | ("Torres Strait Islander*" or Maori* or "American Indian*" or "Alask* Nativ*" or "Nativ* Alask*" or "Nativ* Hawaiian*" or "Hawaii* Nativ*" or "Nativ* Americ*" or "Americ* Nativ*" or "Americ* Samoa*" or "Samoa* Americ*" or Eskimo* or Inuit* or Aleut* or Metis).mp. |
| 3 | ("Torres Strait Islander*" or Maori* or "American Indian*" or "Alask* Nativ*" or "Nativ* Alask*" or "Nativ* Hawaiian*" or "Hawaii* Nativ*" or "Nativ* Americ*" or "Americ* Nativ*" or "Americ* Samoa*" or "Samoa* Americ*" or Eskimo* or Inuit* or Aleut* or Metis).mp. |
| 4 | Stress Disorder*.mp. |
| 5 | ("PTSD" or "posttrauma*" or "post-trauma*" or "post trauma*" or "stress disorder*").mp. |
| 6 | ("out of home care").mp. |
| 7 | (((History or histories or historic* or survivor* or continuity or cycle* or discontinuity or disclos* or recover* or unresolved or past or cultur* or coloni* or generation* or intergeneration* or inter-generation*) adj2 (abus* or adversity or maltreat* or neglect* or posttrauma* or post-trauma* or stress or trauma* or violen*)) not ("physical trauma" or "head trauma" or "traumatic brain injury")).mp. |
| 8 | Medline: Health Services Indigenous/  PsycINFO: Health Care Services/  Embase: Health Service/ |
| 9 | (Service* or approach* or strateg* or program* or framework* or practice* or tool* or casework*).mp. |
| 10 | ((trauma* or cultur*) adj2 inform*).mp. |
| 11 | (healing or wellbeing or "well being" or "strength based" or holistic or integrat*).mp. |
| 12 | 1 or 2 or 3 |
| 13 | 4 or 5 or 6 |
| 14 | 7 or 8 or 9 or 10 |
| 15 | 12 and 13 and 14 |
| 16 | limit 15 to (english language and yr="2012 -Current") |

**Informit**: **983** **results on July 19, 2023, updated July 2, 2025**

| 1 | trauma OR violence OR abus* OR "out of home care" OR PTSD OR "posttrauma*" OR "post-trauma*" OR "post trauma*" OR "stress disorder*" OR "inter*generation*" OR adversity OR maltreat* OR neglect* |
| --- | --- |
| 2 | Service* OR approach* OR strateg* OR program* OR framework* OR practice* OR tool* OR casework* OR "Trauma inform*" OR "cultur* inform*" OR healing OR wellbeing OR "well being" OR "strength based" OR holistic OR integrative |

### **Table A2.** Relevance and quality scoring criteria

| Relevance | Quality |
| --- | --- |
| 1. Trauma specific service for Aboriginal and Torres Strait Islander people 2. Service providing support for Aboriginal and Torres Strait Islander people experiencing traumatic events (family violence, child sexual abuse, Out of Home Care, and natural disasters) 3. Other (general healing services, social and emotional wellbeing) | 1. Evaluation reports published post-2012 2. Evaluation reports published pre-2012 or organizational reports lacking sufficient detail 3. Annual reports published post-2012 4. Annual reports published pre-2012 or annual reports lacking sufficient details 5. Detailed descriptions of program/service only 6. Outline of programs/services only |

### **Table A3.** Evaluation indicator definitions

| Indicator | Definition |
| --- | --- |
| Effectiveness | Extent to which services and programs achieve their goals. In this context, this includes positive outcomes in the domains of social and emotional wellbeing (e.g., health and wellbeing, employment, education attainment, connection to kin and culture), contact with child protection services or the justice system and program cost-effectiveness. |
| Acceptability | Extent to which a service or program is perceived as suitable, appropriate and useful by service users and/or providers. This includes service user feedback (such as satisfaction surveys), engagement, attainment and dropout rates and testimonials. |
| Feasibility | Practicality and viability of implementing a service or program. This includes consideration of barriers and enablers to service implementation, acceptability and effectiveness. |

## Appendix 2: Exemplar services and programs

### Table A4. Exemplar program: Cultural Healing Programs

| Location | Victoria | |
| --- | --- | --- |
| Setting | Not-for-profit, VACCA, ACCO | |
| Operational status | Programs completed | |
| Target population | Stolen Generation survivors of institutional child sexual abuse; children and young people in out-of-home care | |
| Aim | To address the untreated healing needs of Stolen Generational survivors from childhood institutional sexual abuse and cultural abuse | |
| Framework | Cultural Therapeutic Ways (Wise et al., 2024) | |
| Guiding principles | - Context and knowledges (colonisation history, institutional abuse, Aboriginal knowledges have been denigrated and denied, empowerment through co-design) - Cultural connection and healing (identity, safety) | |
| Relevant programs | 1. Yorta Yorta Cultural Healing Camp 2. Wurundjeri Women's Healing Program 3. Dja Dja Wurrong Community Healing Gathering 4. Dja Dja Wurrong Women's Cultural Healing Gathering 5. Narrun Yana art collective | |
| Core components | Across all programs   - Ceremonies: Welcome to Country, smoking ceremonies, closing ceremony, song and dance - Cultural practices: arts and crafts, painting canvases, coolamons, clap sticks, boomerangs, and rocks, basket weaving, making nulla nullas and boondies, Koorie jewelry making, creating possum skin cloaks - Explore and strengthen identity and connection to community: tracing family history and cultural tours - Self-care, healing and wellbeing activities - Sharing of knowledge of past policies, laws and history of removal, impact of removal and losses. - Storytelling and yarning: yarning circles, elders' yarns, cultural storytelling with cultural custodians. - Sharing of meals - Transportation - The Narrun Yana art collective was designed by young people for young people, and also included artistic training, mentoring and business skill development. | |
| Staffing | Four facilitators (three Aboriginal, one non-Aboriginal). The non-Aboriginal facilitator was strongly connected to Aboriginal community and had worked in community for over 35 years. Facilitators trained in either social work or family therapy | |
| Evaluation Design | **Design:** Qualitative, peer-review papers (Black et al., 2019, 2024)  **Aboriginal governance, design and execution in the evaluation**: Aboriginal involvement and input in the program design, process and evaluation. To ensure integrity of the evaluation process, non-Aboriginal researchers were vouched for by survivors, facilitators, elders and community members.  **Published:** 2019, 2023  **Evaluation period:** Not reported; 2020-2021  **Sample size:** 62; 3  **Age:** Not reported; early- to mid-20s  **Gender:** Not reported; women | |
| Evaluation indicators | Effectiveness | - Participants reported feeling better, finding 'peace and happiness', that it ‘cleared my mind', allowed them 'keep my culture, identity and dignity and who I am', increased - Participants reported positive SEWB impacts, including safety and security, increased self-esteem, growth in relationships and increased confidence. - Significant power in storytelling: sharing, talking and listening to others' stories - Empowerment: participants reported feeling strengthened by their connection to and knowledge of culture. Recognition that their trauma was a consequence of invasion and colonisation was important to feeling empowered. Many participants were empowered to advocate and have their voices heard, and felt empowered by being entrusted as cultural knowledge holders |
|  | Acceptability | - Participants felt safe: support from facilitators, company of other survivors, program conducted on country, ceremony, cultural arts and crafts and focusing on family |
|  | Feasibility | Enablers   - Culturally grounded - Aboriginal-leadership - Program fostered connection and belonging through shared experiences - Centrality of relationships (peer support, elders, community involvement) - Providing holistic support - Qualitative research methods capturing the voices of young people |
|  |  | Barriers: Not reported |
| Other | Funded by Commonwealth and State Governments following advocacy from VACCA | |

### Table A5. Exemplar service: Dardi Munwurro

| Location | Victoria | |
| --- | --- | --- |
| Setting | Not-for-profit, specialist Aboriginal family violence service, ACCO | |
| Operational status | In operation since 2000 | |
| Target population | Aboriginal men who use violence (including men on Family Violence Intervention Orders, those charged with family violence offences in the previous 12 months, court-ordered referrals, and self-referrals from Dardi Munwurro’s prison program) | |
| Aim | To break the cycle of intergenerational trauma in the Aboriginal community and disrupt the patterns of behaviour that can result in domestic violence. These programs support Aboriginal men to recognise their emotions, strengths and their own responsibility by using traditional Aboriginal healing practices | |
| Framework | Not reported | |
| Guiding principles | Not reported | |
| Relevant programs | 1. Men's Healing and Behavioural Change 2. Bramung Jaarn (Journeys Program) 3. Ngarra Jarranounith Place (residential program) | |
| Core components | Healing programs take place in culturally safe setting, such as traditional lands or at Aboriginal and Torres Strait Islander community-controlled locations  **Men's Healing and Behavioural Change:** Weekly/fortnightly men’s counselling group sessions addressing drivers for violence by strengthening cultural connection, developing pride and confidence, and planning a future with healthy relationships in families and communities.  **Bramung Jaarn:** draws on key elements of the Men's Healing and Behavioural Change program to engage and empower young Aboriginal men (aged 10-17 years) with the intention of diverting them from the justice system through group sessions, one-on-one mentoring, and access to strong male mentors and Elders.  **Ngarra Jarranounith Place:** a 16-week intensive residential program that supports at-risk men to strengthen their spirit and culture, adopt positive behaviours, and nurture healthy relationships. Programs individual support, group activities and medically supervised detoxification (where required). Team consists of Aboriginal psychologists, allied health professionals and Family Engagement and Safety workers. | |
| Staffing | **Men's Healing and Behavioural Change:** Each session run by two facilitators and community Elders.  **Bramung Jaarn:** Not reported  **Ngarra Jarranounith Place:** Aboriginal psychologists, allied health professionals and Family Engagement and Safety workers | |
| Evaluation Design | **Design:** Cost-benefit analysis (mixed-methods), independently conducted (Deloitte) (Deloitte Access Economics, 2021)  **Aboriginal governance, design and execution in the evaluation**: not reported  **Published:** 2021  **Evaluation period:** not reported  **Sample size:** 80  **Age:** Not reported  **Gender:** Not reported | |
| Evaluation indicators | Effectiveness | - Improved social and emotional wellbeing (connection to culture, identity and community, improved relationships and taking responsibility for behaviour) - Reduction in substance use (80% pre-program vs 34% following program completion) - 45% increase (33 pre-program to 48 post-program) in men returning to their family/kinship homes or secured own accommodation, with number of men experiencing homelessness reducing by 100% - Improved educational attainment (number of young people engaged in education programs doubled) - Reduced contact with justice system (less likely to be incarcerated or subject to court order) - Improved family violence outcomes (80% reduction in reported family violence incidents following program completion) - Increased rates of employment following program completion - For every dollar invested in the programs, there are 50%-190% return on investments (cost-benefit ratio of 1.5-2.90) |
|  | Acceptability | Not reported |
|  | Feasibility | Enablers   - Positive return on investment |
|  |  | Barriers: Not reported |
| Other | Funded by the Healing Foundation | |

### Table A6. Exemplar service: Deadly Connections Community Services

| Location | New South Wales | | |
| --- | --- | --- | --- |
| Setting | Not-for-profit, community and justice specialist agency, ACCO | | |
| Operational status | In operation since 2018 | | |
| Target population | Aboriginal people, families and communities | | |
| Aim | To break cycles of disadvantage, trauma, child protection and justice involvement | | |
| Framework | - Self-determination - Healing centred engagement - Lived-experience | | - Life course approach - Holistic |
| Guiding principles | - Creation of safety within self and environment - Stability and structure - Programs developed by and for community - Culturally responsive ways to support First Nations people - Caring for community, families, children and individuals - Holistic, practical and culturally responsive programming - Grounded in culture, respect and identity - Collaborative partnerships with community, partners and government - Long term commitment and responsibility to community - Create innovative solutions and opportunities to amplify community's voice and enable Aboriginal-led and community driven solutions - Provision of accessible, fun and pro-social activities | | |
| Relevant programs | 1. Girra Girra Healing Place 2. Deadly Families 3. Deadly young warriors 4. Deadly Futures | | 1. Deadly Pathways 2. Deadly Brothers and Deadly Tiddas |
| Core components | - Education programs - Cultural activities to promote connection within family - Contemporary cultural healing activities - Support service navigation/advocacy - Case management | | - Multimodal programs tailored to suit clients' needs - Men's and Women's groups - Cultural camps - Therapeutic group work - Case advocacy - Mindfulness |
| Staffing | 3 directors and 10 Aboriginal and non-Aboriginal staff members with lived experience in the healing journey, based on a credible messenger model.  Student placements and volunteers (number not reported)  Advisory board (11 members: professionals, Elders and community members) | | |
| Evaluation Design | **All services – social impact report** (Deadly Connections, 2021)  **Design:** Mixed methods, evaluation independently conducted (Huber Social)  **Aboriginal governance, design and execution in the evaluation**: Not reported  **Published:** 2022  **Evaluation period:** 2018 – 2021  **Sample size:** Not reported  **Age:** Not reported  **Gender:** Not reported | | **Deadly families program**  **Design:** Mixed methods, evaluation independently conducted (For-Purpose Evaluations) (Booth, 2020)  **Aboriginal governance, design and execution in the evaluation**: the evaluation plan was co-designed with Aboriginal staff  **Published:** 2020  **Evaluation period:** 3 months in 2020  **Sample size:** 21 interviewees  **Age:** Not reported  **Gender:** Not reported |
| Evaluation indicators | Effectiveness | - Improved connection to identity, which was associated with higher wellbeing scores - Across all programs, wellbeing was 49% higher after 3 months, and 42% high among former clients than people on intake - **Deadly Families**: Clients reported 72% higher overall wellbeing, with the greatest improvement in building and managing healthy relationships, 43% decrease in number of DCJ reports of abuse/neglect, 44% increase in connection to culture, language and country and a 56% increase in positive parenting interactions. It deepened trust and engagement with services, increased agency and confidence when navigating social service systems, empowered Aboriginal people to know their rights, advocate for themselves and their cultural needs. Within the first 12 months of the program, there was an estimated return on investment is $1.02 for every donated/funded dollar - **Breaking the cycle**: Clients reported 41% higher overall wellbeing, with the greatest improvements in holistic wellness, relationships and daily life skills - **Deadly Young Warriors**: Participants feel confident in resilience and sense of community connection | |
|  | Acceptability | - Program highly valued by program participants. 78% of participants in the Deadly Family program reported a positive experience - Participants reported feeling safe and listened to   Rates of engagement (Deadly Families)   - 52% increase in attendance to planned appointments - 51% increase in answering calls - 36% reduction in re-scheduled appointments | |
|  | Feasibility | Enablers   - Flexibility - Cultural safety - Warm, loving and supportive - Commitment to the client - Feeling listened to - Aboriginal staff with lived experience - Realistic, practical support | |
|  |  | Barriers   - Funding limitations have led to casualisation of workforce causing difficulties with retention and stability of staff - Unsustainable data collection methods and client management system with rapid increase in program participant numbers | |

### **Table A7.** Exemplar service: Djirra

| Location | Victoria | |
| --- | --- | --- |
| Setting | Not-for-profit, Community family violence service, ACCO | |
| Operational status | In operation since 2007 | |
| Target population | Aboriginal women who experience violence | |
| Aim | To build capacity to prevent family violence, address the impacts of family violence, and reduce family violence re-victimisation. | |
| Framework | Not reported | |
| Guiding principles | - Culturally safe - Respect the personal and sensitive nature of family violence - Flexible approach focusing on the participants’ social and emotional well-being - Acknowledge history and the impacts of colonisation - Strengths-based | |
| Relevant programs | 1. Sisters Day Out 2. Dilly Bag 3. Dilly Bag: The Journey | |
| Core components | **Sister’s Day Out:** access to services, self-care and wellbeing, access to information and education (in a relaxed, informal environment)  **Dilly Bag Programs:** small groups, overnight stays, weaving, beading (while sharing stories and experiences) | |
| Staffing | Program facilitators are Aboriginal women with over 20 years experience working in Victorian Aboriginal communities who are known and trusted by community members | |
| Evaluation Design | **Design:** Mixed-methods, independently conducted (agency not reported), (Aboriginal Family Violence Prevention And Legal Service Victoria, 2014)  **Aboriginal governance, design and execution in the evaluation**: Special care was taken to avoid evaluation methods that were deemed to represent ‘white system’ tools  **Published:** 2021  **Evaluation period:** 2007-2014  **Sample size:** interviews with 55 program participants; number of service providers interviewed not reported; number of participant satisfaction surveys completed not reported  **Age:** Not reported  **Gender:** 100% women  Information has also been extracted from the 2024 Annual Report (Djirra, 2024) | |
| Evaluation indicators | Effectiveness | - Enhanced participant’s self-esteem and well-being - Strengthened friendships/relationships/connections within the community and increased community networks (reduced isolation) - Strengthened individual participant’s resilience and the community’s ability to address family violence - Increased participant’s knowledge and understanding of family violence - Increased participant’s awareness of support and legal services, both Aboriginal specific and mainstream (e.g., some participants become clients of FVPLS Victoria following the workshops). - Since the first Sister’s Day Out workshop, 88 participants had been referred to other services (59 referrals to legal service, 29 to other services). This data is likely to be under-representative. |
|  | Acceptability | - Participant and service provider feedback was very positive - Participants reported feeling safe, relaxed and comfortable   Rates of engagement   - Since 2007, 84 Sister's Day Out workshops with 6,078 Aboriginal women have been conducted - Since 2010, 16 Dilly Bag workshops with 166 Aboriginal women have been conducted - 2023-2024: 132 women attended 14 Dilly Bag workshops; 1024 women attended 14 Sisters Day Out workshops |
|  | Feasibility | Enablers   - Incorporation of cultural elements to facilitate safety within program design - Welcoming environment: venues are easily accessible, peaceful and purposefully decorated; promotion of program through community networks - Trust: employment of Aboriginal women, opportunities to speak with mainstream service providers - Language: using the community’s language rather than mainstream terms e.g., ‘day out with sisters’ vs workshops/activities - Power relationships with mainstream service providers inverted as they participate by invitation only; participants able to approach service providers in relaxed environment |
|  |  | Barriers   - Lack of childcare during program activities - In 2012, a reduction in Federal funding resulted in a loss of $150,000 that was used to fund the programs. Since then, the programs have been funded by ad-hoc limited grants. Insecure funding drains considerable resources from the organisation due to time spend identifying and applying for funding. - Data collection points for robust referral data need to be strengthened. |
| Other | - Funded by the Mallee District Aboriginal Services, Commonwealth Department of Social Services, Department of Justice Victoria, Department of Human Services Victoria and Relationships Australia. - Limited data, including referral data from all services, was a barrier to evaluation. | |

### Table A8. Exemplar service: Kornar Winmil Yunti (KWY) Aboriginal Corporation

| Location | South Australia | | |
| --- | --- | --- | --- |
| Setting | Not-for-profit, ACCO | | |
| Operational status | In operation since 2011 | | |
| Target population | Aboriginal families experiencing family violence and are at risk of having children removed and/or having a family member enter the criminal justice system due to perpetrating family violence | | |
| Aim | To reduce family violence in the Aboriginal and Torres Strait Islander communities, and as a result reduce the number of children entering the out of home care and juvenile justice systems by breaking the cycles of family violence | | |
| Framework | Holistic Family Hub Model | | |
| Guiding principles | - Cultural ways of healing and engagement - Safe space to encourage reconnection with culture, spirit and community | | |
| Relevant programs | 1. Stronger, Safer Families Outreach Hubs 2. Aboriginal women’s safety contact service 3. Taikurtirna Tirra-Apinthi | | 1. Intensive Family Support (Ana Wardli – Towards Home, Walking Together) 2. My Journey 3. Healing by Art Program |
| Core components | - Referral process - Intake/assessment - Case plans/risk assessment - Workshops - Counselling - Group work | | - Educational therapy - Financial counselling - Activities for children (dance, music, art, narrative, community events, cultural camps) |
| Staffing | - 3 men’s family violence counsellors - 3 women’s family violence counsellors - 1 social worker - 2 children’s trauma counsellors - 1 operations manager/practitioner supervisor - 1 administration officer | | - CEO and ‘many counsellors’ are Aboriginal people. All staff experienced in working with Aboriginal communities and are ‘exceedingly’ culturally competent. - Staff able to choose therapy modalities most comfortable for them. - All staff trained in CBT |
| Evaluation Design | **Design:** Mixed-methods, independently conducted (RAND) (Cahill et al., 2021)  **Aboriginal governance, design and execution in the evaluation**: Assistance from Inside Policy, an Aboriginal-owned organization in setting up the evaluation  **Published:** 2021  **Evaluation period:** 2018-2020  **Sample size:** Not reported  **Age:** Not reported  **Gender:** Not reported | | |
| Evaluation indicators | Effectiveness | No outcomes reported in the evaluation report as after over two-years of serving clients, the data collected by KWY was not sufficient to enable a rigorous evaluation to be conducted | |
|  | Acceptability | Not reported | |
|  | Feasibility | Enablers   - Organisation and staff strongly believed in the value of their program - Staff had experience working with Aboriginal populations and were exceedingly culturally competent - Program was adapted to address staffing challenges - Staff able to choose modality of therapy most comfortable for them, all were trained in CBT. | |
|  |  | Barriers   - Difficulties maintaining contact with clients (as they changed phone numbers regularly) - Lack of culturally appropriate measure for child risk assessment - Lack of central database (excel was not efficient) - Hard to adhere to the holistic family model due to staff shortages in remote areas | |
| Other | - Funded by the Department of the Prime Minister and Cabinet Third Action Plan - Institutionalising service-wide data collection is a significant undertaking - Evaluation timeline limited the period during which actual program implementation could be observed | | |

### Table A9. Exemplar service: Mpwelarre Health Aboriginal Corporation

| Location | Northern Territory | | |
| --- | --- | --- | --- |
| Setting | Not-for-profit, primary health care service, ACCHO | | |
| Operational status | In operation since 1973 | | |
| Target population | Aboriginal and Torres Strait Islander people living in a remote communities | | |
| Aim | To provide holistic and culturally appropriate primary health care for social and cultural wellbeing, mental health and community connectedness | | |
| Framework | Not reported | | |
| Guiding principles | - Community engagement with the project - Response to identified community needs - Delivery and responsibility for the project sits with Aboriginal people - Culturally valid understandings must shape the provision of services | | |
| Relevant programs | Social and Emotional Wellbeing Service | | |
| Core components | - Individual, family and large group activities - Individual counselling - Family therapy - Narrative therapy - Play therapy, sand play | | - Traditional healing - Cultural activities (e.g., men's dancing) - Community engagement activities - Community education |
| Staffing | Two Aboriginal Family Workers (AFW) that reside in the community, two non-Indigenous counsellors that spend significant time residing in the community. One AFW is also a traditional healer. SEWBS staff work with other health and allied health professionals from the Health Service and other organisations. | | |
| Evaluation Design | **Design:** Cross-sectional qualitative study (Carey, 2013)  **Aboriginal governance, design and execution in the evaluation**: participants offered opportunity to review transcripts; over a 12-month period, the principal researcher spent time in community to develop and build relationships, which informed the design of the research  **Published:** 2013  **Evaluation period:** Not reported  **Sample size:** 21 (service providers, service participants and referrers)  **Age:** Not reported  **Gender:** 62% male | | |
| Evaluation indicators | Effectiveness | - Reductions in smoking, alcohol, self-harm - Increased engagement and sense of value - Provision of localised response to significant social and interpersonal problems has resulted in reduction of these problems | |
|  | Acceptability | - Participants reported that the program has been an effective response to significant community problems | |
|  | Feasibility | Enablers   - Strong teamwork between SEWB service staff, colleagues in wider service and other local groups. - Working flexibly - Having a trusted person to talk to - Targeting multiple levels of change | |
|  |  | Barriers   - Demands of the service leave providers overwhelmed and exhausted - Communication barriers - Difficulty establishing, maintaining and promoting access - Difficulty recruiting staff - Lack of clear decision-making pathways - Issues with funding: Funding from multiple sources with lack of funding sustainability; funding decisions being made by non-Indigenous people focusing on a deficit model (rather than strengths-based) - Reporting requirements difficult to balance with the role of the service. | |
| Other | - Community identified the need for the evaluation, stating that it was integral to accountability | | |

### Table A10. Exemplar program: Murri School Healing Program

| Location | Queensland | | |
| --- | --- | --- | --- |
| Setting | Not-for-profit, school, Indigenous owned and controlled | | |
| Operational status | Established 2012, unclear if in operation | | |
| Target population | Aboriginal and Torres Strait Islander students and parents | | |
| Aim | To assist young Aboriginal and Torres Strait Islander people to heal from their distress and prevent the continuing transmission of trauma | | |
| Framework | Trauma-aware and healing-informed | | |
| Guiding principles | - Connection to culture - Improved family relationships | | - Improved service coordination - Improved physical health |
| Core components | - Healing camps - Healing circles - Use of country to support increased cultural connection - Outreach support - Building pride and identity through cultural activities and experiences - Mentoring and personal development programs | | - Counselling and therapeutic support - Health service provision - Case management - Service coordination (e.g., DOCS, Centrelink, Housing, Evolve, Kummara, Kurbingui and Distance Education) |
| Staffing | Family support workers, psychologists, medical and allied health professionals and trauma-informed teachers | | |
| Evaluation Design | **Design:** Cost-benefit analysis (mixed-methods), independently conducted (Deloitte) (Deloitte Access Economics, 2017)  **Aboriginal governance, design and execution in the evaluation**: Not reported  **Published:** 2017  **Evaluation period:** 2012-2016  **Sample size:** 161 survey respondents  **Age:** Not reported  **Gender:** Not reported | | |
| Evaluation indicators | Effectiveness | - Improved social and emotional wellbeing (a reduction of ‘abnormal’ and ‘borderline’ scores in the Strengths and Difficulties Questionnaire) - Reduced contact with child protection services (30% reduction in OOHC placements, 19% reduction in contact with CPS) - Reduced contact with justice system (14% reduction in incarcerations) - Improved education attainment (higher than average Year 12 completion rates and school attendance rates) - Improved connection to culture: 77% of young people reported that the camp activities helped them strengthen their connection to culture - Benefits of the healing program were calculated to be worth $28,248 per student - For every dollar invested in the program, there are $8.85 return benefits | |
|  | Acceptability | Rates of engagement (Jan 2016-June 2016)   - 405 children and 129 family members undertook counselling - 230 young people and 180 adult family members took part in healing activities | |
|  | Feasibility | Enablers   - Employment of Aboriginal and Torres Strait Islander staff - Positive return on investment | |
|  |  | Barriers: Not reported | |
| Other | - Part of the Healing Foundation’s Intergenerational Trauma Initiative funding - The Healing programs at Brewarrina Central School and Bourke High School are based on the Murri Model - The Murri School is also known as The Aboriginal and Islander Independent Community School | | |

### Table A11. Exemplar service: Marninwarntikura Women's Resource Centre

| Location | Western Australia | | |
| --- | --- | --- | --- |
| Setting | Not-for-profit, community service, ACCO | | |
| Operational status | In operation since 2003 | | |
| Target population | Aboriginal women and children | | |
| Aim | To strengthen families through the journey of healing from intergenerational and early life trauma, family violence, grief and loss | | |
| Framework | Marroorryawarrani Ngambirriyawarrani Yoowarnia (Gathering, Caring and Nurturing Together as One) (Marninwarntikura Women's Resource Centre, 2022) | | |
| Guiding principles | - Knowing history (decolonisation) - Making connections - Creating safe spaces - Connection to Country - Accessing the right kind of support - Self-care - Collective care - Advocacy Culture at the centre "culture is healing" - Working with people where they are at - Showing people what 'good' looks like - Family and community led - Leadership - Flexibility | | - Build relationships and partnerships Caring, nurturing, empathy, understanding - Strengths-based - Privileging family voices - Commitment (being there for the long journey) - Accountability - Informed by emerging knowledge, lived experience, and grounded in healing - Healing and trauma aware practices - Guided by cultural authority and women’s voices - Supporting people to heal in their own way. |
| Relevant programs | 1. Baya Gawiy Buga Yani Jandu Yani U 2. Crisis response (Family Violence Prevention Legal Unit) 3. Marulu 4. Marnin Studio 5. Bigiswun Social and Emotional Wellbeing program | | |
| Core components | - Sharing information - Creating connection - Supporting autonomy and choice - Healing and trauma aware practice – integration into all aspects of organisation - Acknowledging feelings - Responding to and supporting people to navigate government policy and systems - Importance of relationships – building trust - Advocacy both individual and systemic | | - Referral integrated services across Marninwarntikura and external referral - Leadership and modelling - Culturally informed practice - Language learning - Building confidence and self-esteem, positive mental health and wellbeing - Embedding cultural parenting, practices, language - Yarning - Nurturing self and others |
| Staffing | Indigenous and non-Indigenous staff members, specific staffing composition unclear. Legal service includes a counsellor and social worker | | |
| Evaluation Design | **Design:** Mixed methods, independent review of the Women’s Shelter (Moreton Consulting) (Pearce, 2017)  **Aboriginal governance, design and execution in the evaluation**: Moreton Consulting (now called Burbangana Group) is Indigenous owned and controlled; inclusion of a project advisory group to guide the co-design process  **Published:** 2017, 2021, 2023, 2024  **Evaluation period:** Not reported  **Sample size:** Not reported  **Age:** Not reported  **Gender:** Not reported  Information has also been extracted from the 2021, 2023 and 2024 Annual Report (Marninwarntikura Women's Resource Centre, 2021, 2023, 2024) | | |
| Evaluation indicators | Effectiveness | - Various recommendations came from the 2017 review of the Women’s Shelter, including: - changing the model of care to a strengths-based wrap around support for women and children - improving staffing by employing more permanent positions and providing opportunities for professional development and training - create a culturally safe and friendly environment; - increase opportunities for outreach - Evidence from the Annual Reports indicates that these recommendations have been taken on board, and that overall the shelter is running well. - The team have incorporated a more trauma-informed understandings and approaches to the physical spaces in which women stay. For example, they have brought more greenery into the courtyard and common areas, reinvigorated our outdoor furniture, upgraded the bathroom areas, rethought language and information displays replacing what wasn’t trauma informed with art created by women staying in the Shelter. These changes were reported to enhance the well-being of the women and children staying in the shelter. - Efforts made to shift perceptions of domestic violence among shelter users by providing tools to identify early warning signs of domestic violence and develop effective safety plans - The reports also indicates that the team have implemented a Mobile Outreach Program and have a more stable staffing roster (including transitioning casual staff into full-time roles) | |
|  | Acceptability | Not reported | |
|  | Feasibility | Enablers   - Evidence from the Annual Reports (across all programs) - Employing local staff on Country - Investment in workforce skills development and capacity building - Regular pickup/drop off bus service - Use of a inquiry-based learning model. | |
|  |  | Barriers   - Evidence from the 2017 review on the Women’s Shelter: - Outreach activities limited by funding - Staffing complications (majority of staff were in casual positions) - The shelter had a poor reputation - The shelter did not provide enough wrap-around support - Poor cultural sensitivity from staff | |
| Other | - Linked to Healing Foundation Trauma Education Programs - Community identified the need for the evaluation, stating that it was integral to accountability - Funding for the expansion of the Family Violence Prevention Legal Unit from Department of the Prime Minister and Cabinet Third Action Plan. Although MWRC was included in the Final Report by RAND (Australia's Third Action Plan of the National Plan to Reduce Violence Against Women and Their Children, Priority Area 2), as the funding was limited to the appoint of a counsellor and social worker, MWRC were unable to show evidence of program improvement. Staff did report anecdotally that clients were doing better overall | | |

### Table A12. Exemplar program: North Australian Aboriginal Justice Agency (NAAJA) Healing Program

| Location | Northern Territory | |
| --- | --- | --- |
| Setting | Not-for-profit, legal service, Aboriginal governance | |
| Operational status | In operation between 2017 to 2018 | |
| Target population | Aboriginal youth with contact with justice system | |
| Aim | To strengthen cultural identity, build skills and re-establish positive relationships | |
| Framework | Trauma-aware and healing-informed approaches | |
| Guiding principles | Not reported | |
| Core components | - Focus on future goals and engagement in employment and training - DRUMBEAT and yarning group therapeutic sessions (individual and group) - Weekly activities to strengthen cultural identity - Young fathers took part in a parenting program - Parents of young people offered support to care for their children’s trauma and to re-establish positive relationships following their release | |
| Staffing | NAAJA youth support workers, a Youth Engagement Counsellor based at SEWB branch of Danila Dilba Health Service, a Youth team coordinator | |
| Evaluation Design | **Design:** Document review of biannual Performance Reports (planned yarning circles were not conducted due to COVID-19 restrictions) (Prince, 2021)  **Aboriginal governance, design and execution in the evaluation**: Not reported  **Published:** 2021  **Evaluation period:** 2017-2018  **Sample size:** 70  **Age:** Not reported  **Gender:** 100% male | |
| Evaluation indicators | Effectiveness | - Improved social and emotional wellbeing of children and young people - Improved sense of belonging and connection to culture - Improved resiliency of children and young people (including skills in managing conflict and enhanced help-seeking behaviours, securing employment) - Improved relationships/stronger connections between young people and their families - Improved service coordination for children and young people, and families. - Workforce development: Complex Case Clinic training for NAAJA team members (completed 5 training sessions during the program) |
|  | Acceptability | - Participating young males and their families reported 90% satisfaction rating with the project - Pre-release relationship building excellent, safe, and provided a trusting platform to continue working with the young person and their family post-release |
|  | Feasibility | Enablers   - Use of natural spaces, such beaches and parks to conduct therapeutic sessions - Partnerships and collaboration with teams from other organisations including Community Corrections, Youth Outreach and Engagement teams and NAAJA youth lawyers. - Complex Case Clinics were a key success factor. |
|  |  | Barriers   - Lack of physical space in the detention centre to conduct therapeutic conversations - Limited access to the detention centre - Personnel changes in partner organisations - Collaboration with some key stakeholders - Introduction of the Territory Families based Youth Outreach and Engagement Officers in early 2018 shifted support away from an Indigenous trauma-aware and healing-informed service |
| Other | Funded by the Healing Foundation, collaboration between North Australian Aboriginal Justice Agency, The Healing Foundation, and Relationships Australia | |

### Table A13. Exemplar service: Neami National

| Location | Victoria | | |
| --- | --- | --- | --- |
| Setting | Not-for-profit, mainstream mental health provider | | |
| Operational status | In operation since 2013 | | |
| Target population | Aboriginal people experiencing mental illness and poor social and emotional wellbeing, with a history of homelessness | | |
| Aim | To address the physical and mental health needs of individuals with complex histories of trauma and neglect and to remove barriers to service access by creating safe and trustworthy service systems | | |
| Framework | Wadamba Wilam Practice Approach (Theory of Change)   1. Foundation activities (see guiding principles) 2. Relational activities and immediate changes (Basic needs are met; hooks are offered in relation to need; the service meets people where they are at; not shaming; being with people in crisis; slow and flexible assessment; tools used at the right time, over time; early use of service systems; staff look for therapeutic windows; permission to spend time to build relationship; persistence - workers come back again; staff seek to understand rather than react; staff hold issues and come back to them at the right time) 3. Foundational outcomes (sense of hope is generated; workers are trusted; people are understood within a context of culture and trauma, informed by their social historical context) 4. Intermediate outcomes (flexible & responsive access; service is accepted and valued by community; Consumers choose to strengthen connections in relation to their wellbeing; Consumers can link with and use systems towards wellbeing; People feel safe to engage in healing activities) 5. End of program outcomes: Aboriginal people feel increased sense of their wellbeing, strength and connection 6. Long term goals: Holistic social and emotional wellbeing for Aboriginal people | | |
| Guiding principles | - Interdisciplinary team - Cultural responsiveness and culturally safe practice - Alignment with the 9 principles of the Social and Emotional Wellbeing - Family-centred practice - Strengths-based approach - Trauma-informed care - Culturally appropriate tools - Care coordination | | - Continuity of care - Interdisciplinary team approach - Discretionary budget - Long term support to build trusting relationships, with continued support after basic needs are met and soft endings to allow consumers to build gradually towards independence. |
| Core components | - Holistic mental health treatment and psychosocial support with a focus on trauma recovery and improving social and emotional wellbeing - Specialist Alcohol and Other Drug treatment and support - Case management and care co-ordination, which involves facilitating engagement with necessary social services and community resources such as cultural groups and camps. - Advocacy and support for navigating the myriad systems involved in consumers’ care, including housing - Promoting and facilitating engagement - Liaison, education and involvement with the identified family to support and empower the consumer. - Activities to support connection to culture, including Men’s Camps, time spent on Country, attendance at cultural events, provision of art supplies, among others. | | |
| Staffing | - Aboriginal and non-Aboriginal staff (non-Aboriginal staff have completed cultural responsiveness training, participate in cultural mentoring) - Outreach team consists of healthcare professionals representing Victorian Aboriginal Health Service, Neami National, Uniting Care ReGen Alcohol and Other Drug Service, and the Northern Area Mental Health Service. - The interdisciplinary team includes an Aboriginal Social and Emotional Wellbeing support worker, a Nurse Practitioner, a Senior AOD Clinician, two Community Rehabilitation Support Workers, a Consultant Psychiatrist and a Service Manager. | | |
| Evaluation Design | **Design:** Mixed methods, organizational impact report (Chiera, 2021)  **Aboriginal governance, design and execution in the evaluation**: Not reported  **Published:** 2021  **Evaluation period:** 2016-2020  **Sample size:** 48  **Age:** Not reported  **Gender:** Not reported | | |
| Evaluation indicators | Effectiveness | - 81% of consumers are housed in sustainable tenancies, with 69% of consumers sustaining these tenancies for over 12 months. - 61% decrease in the number of inpatient psychiatric admissions post-referral. The average yearly psychiatric admissions reduced from 1.5 per year per person, to 0.1 per year per person. - 67% of consumers have experienced an increase in their meaningful activity, shifting from 73% of consumers engaging in survival activities only at service entry. - 72% of consumers have reduced both their use of alcohol and ice. 67% of consumers have reduced their use of opioids. - 49% of consumers reported increases in social connections. - 52% of consumers had a reduction in criminal offending. - 73% of consumers had an increase in engagement with medical support, with 44% of these having enough engagement to meet their needs. - 60% of consumers were connected with a regular GP. - 6% of consumers have an Involuntary Treatment Order (ITO) in place, down from 20% of consumers with an active ITO on referral. | |
|  | Acceptability | - Long term support allows trust and rapport to be meaningfully established | |
|  | Feasibility | Enablers   - Interdisciplinary team bring a range of specialty skills that can be utilised to meet complex needs with immediacy. - Intensive assertive outreach and visible presence in the community. - Interagency collaboration overcomes service disconnection. - Low case numbers and a high ratio of staff to consumers ensures staff have adequate time and collegial support to provide the care required to manage complex needs - Intensive assertive outreach, flexibility and responsiveness facilitate workers to overcome service access barriers and to support consumers when in crisis. - A foundational understanding of the nine-guiding principles of social and emotional wellbeing is applied to all aspects of the program. - Culturally appropriate tools, including the Aboriginal Resilience and Recovery Questionnaire (ARRQ) and the International Trauma Questionnaire, are utilised to facilitate meaningful and respectful assessment processes. - The right staff are employed and they behave in a culturally competent and safe manner. They are trauma-informed, understand the impacts of trauma, understand trauma behaviours, and utilise strengths-based interventions to improve people’s wellbeing. - The service works with multiple family members at once, which supports healing to multiple generations and strengthens connections to community and family. - Staff ensure care coordination which supports consumers to navigate various parts of the service system, sharing knowledge and resources with intersecting services to promote effective interventions and avoid re-traumatisation of consumers. - Continuity of care supports the maximising of available interventions, to promote meaningful co-ordination of services and discharge planning. - Workers continue to provide support to consumers after basic needs, such as housing, are met. This supports consumers to make the profound shift from surviving to thriving. - A slow exit process whereby support is provided with less frequency and intensity allows for consumers to build up independence. Quick re-entry to the service if the need arises, without any formal intake process, also allows for consumers to quickly re-engage with their trusted supports to avoid a severe decline in social and emotional wellbeing - Discretionary budget to meet consumer needs. - Trusting relationships established within the community through working collaboratively with ACCHOS and ACCOs | |
|  |  | Barriers: Not reported | |
| Other | - Initially federally funded under the “Breaking the Cycle” initiative, with additional funding to mid-2021 from the Victorian Department of Health and Human Services - The idea of success is not defined by the attainment of broad goals, but rather that success and healing is unique to each person, and better represented by distance travelled than final outcomes | | |

### Table A14. Exemplar service: Ngaanyatjarra Pitjantjatjara Yankunytjatjara Women’s Council

| Location | NPY region (Western Australia, South Australia, Northern Territory) | | |
| --- | --- | --- | --- |
| Setting | Non-governmental, not-for-profit, provision of human services, ACCO | | |
| Operational status | In operation since 1993 | | |
| Target population | Aboriginal and Torres Strait Islander people in remote desert communities | | |
| Aim | To support all Anangu, especially women and children, to have a good life, guided by culture and sound governance, through the collective agency of women. | | |
| Framework | **NPYWC (whole service)**   - Piluntjungku: Peaceful and calm - Ngapartji ngapartjiku kulira iwara wananma tjukarurungku: Respect each other and follow the law straight - Kututu mukulyangku: Kind-hearted - Kunpungku: Strong - Kalypangku: Conciliatory - Tjungungku: United   **Uti Kulin Tjaku Watiku Project: Uti Kulintjaku Iwara** (Togni, 2018; Togni, 2017)   - Thinking work - Emotional work - Supportive work - Iterative learning, reflection and evaluation | | |
| Guiding principles | - Anangu culture, governance and agency - Operational excellence | | - Evidence-based practice - Strong relationships - Anangu-led voice - Financial sustainability |
| Relevant programs | 1. Youth Services 2. Child & Family Wellbeing Service 3. Tjungu Aged & Disability Service 4. Domestic & Family Violence Service (including **Uti Kulin Tjaku Watiku Project**) 5. Tjanpi Desert Weavers Social Enterprise 6. Ngangkari Traditional Healing | | |
| Core components | **Uti Kulin Tjaku Watiku Project**   - Anangu-led collaboration - Safe space to learn, think and share ideas - Strengths-based approach - Aligned with national strategies for family violence prevention in Aboriginal communities   **Activities included:** workshops (with a focus on trauma, the effects of trauma, family violence, responding to and healing from trauma, key mechanism for cross-cultural learning and knowledge exchange; they draw on Anangu and Western knowledge); resources (strengths-based narratives to support family violence prevention, including videos, a toolkit, t-shirts, posters, postcards); intergenerational camps | | |
| Staffing | **Uti Kulin Tjaku Watiku Project**   - Aboriginal men included ngangkari, health practitioners, rangers, carers, artists and leaders of Aboriginal Corporations (e.g. Nganampa Health) - Non-Aboriginal men included mental health professionals, interpreters, project staff and evaluator - Participants were invited by Uti Kulintjaku women, emphasising relationships and leadership in Anangu culture | | |
| Evaluation Design | **Design:** Mixed methods using a developmental evaluation approach (Togni, 2019). Independent evaluation, with the non-Indigenous evaluation team with experience working with Aboriginal people and organisations in Central and northern Australia. The lead evaluator has worked as the evaluator with the women’s Uti Kulintjaku Project team since 2013.  **Aboriginal governance, design and execution in the evaluation:** The evaluation was conducted within an evaluation framework developed through NPYWC in the 1990s to support meaningful evaluation.  **Published:** 2019  **Evaluation period:** 2016 – 2019  **Sample size:** 36 interviews with stakeholders (date not reported), evaluation reflection sessions with the team in 2018 and 2019, reflections at the end of each workshop, analysis of Uti Kulintjaku Watiku Project workshop notes and participant observation at each workshop.  **Age:** Not reported.  **Gender:** Not reported. | | |
| Evaluation indicators | Effectiveness | - Improved ability to describe, understand and respond to personal challenges - Increased knowledge about trauma, its effects on the brain and trauma recovery with greater capacity to articulate complex trauma-related concepts - Participants recognised the breadth and significance of what they learned - Intergenerational learning occurred, with younger men learning from senior men - Participants reported feeling happy and uplifted through sharing positive stories - Men recognised their own capacity to contribute meaningfully - Strengthened relationships with the younger generation - Increased confidence and ability to share knowledge at camps | |
|  | Adaptability | - Fewer attendees at camps than anticipated. However for those who did attend camps, there was good engagement - Initial caution shifted to enthusiasm, with more men wanting to volunteer - Workshops provided a safe space for deep thinking, learning and sharing ideas - Program supported men to engage with issues important to their families and communities - Calm, peaceful environment helped participants focus and think clearly - Participants valued the workshop learning spaces - Knowledge was shared and respected equally among participants - Trusting relationships developed between participants and team members - Participants valued engagement with non-Aboriginal mental health professionals | |
|  | Feasibility | Enablers   - Strength-based approach grounded in a framework (Uti Kulintjaku Iwara) with Anangu leadership - The program was developed by Anangu and led by Anangu for Anangu people - Alignment with national strategies - Conducts its activities primarily in Pitjantjatjara and Ngaanyatjarra (first) languages; - Draws on Anangu and Western evidence-based knowledge - Focusses on activities to strengthen cultural identity and connection - Simplifies men’s stories of resistance to violence. - Includes ongoing evaluation and iterative learning. - Community-led, holistic, strengths-based, trauma informed and grounded in Aboriginal culture and knowledge. - Many of the stakeholders involved in the Project have highlighted the “real hope, energy, commitment and drive” | |
|  |  | Barriers: Not reported | |

### Table A15. Exemplar program: Our Men Our Healing Project

| Location | Northern Territory | | |
| --- | --- | --- | --- |
| Setting | Not-for-profit, Indigenous controlled organization | | |
| Operational status | In operation between 2013 and 2015 in three communities: Maningrida, Ngukurr and Wurrumiyanga | | |
| Target population | Aboriginal and Torres Strait Islander men in remote communities | | |
| Aim | To strengthen, support and empower Aboriginal men | | |
| Framework | - Education: Men taking their place as strong learners and teachers - Employment: Men taking their place as strong providers - Health: Men taking their place as strong men - Identity: Men being stronger in themselves and their place as leaders | | - Law: Men taking their place as positive role models - Relationships: Men taking their place as nurturers - Resources: Men being empowered to be strong in various roles - Safety: Men taking their place as protectors |
| Guiding principles | - Combine western methodologies and Indigenous cultural healing - Understanding the impact of colonisation and transgenerational trauma, and grief in the local community - Build individual, family and community capacity - Incorporate strong evaluation frameworks, communications plans and performance monitoring mechanisms. - Require community readiness, capacity and collaboration - Are supported by effective partnerships across the local service sector - Are embedded in strong Indigenous led organisations with demonstrated healing leadership or other culturally safe, trauma-informed organisations - Driven by local leadership - Have a developed evidence base and theory base | | |
| Core components | - Counselling (e.g., Men’s counselling group) - Family support - Advocacy and cultural brokerage - Case management and coordination - Day trips / activities (e.g., cultural education, fishing trips) | | - Group programs (e.g., the Men's Shed) - Yarning groups - Community events and celebrations (end of year celebrations) - Overnight camps |
| Staffing | - Wurrumiyanga: 3 First Nations staff employed (2014), 2 in 2015. - Maningrida: 6 First Nations staff employed (2014), only 1 in 2015. - Ngukurr: 1 First Nations staff employed in 2014 and 2015. - Men’s group meeting facilitator: Local community member with lived experience of incarceration, violence, healing and participation in healing program. | | |
| Evaluation Design | **Design:** not reported, independent evaluation (Social Compass) (The Healing Foundation, 2015)  **Aboriginal governance, design and execution in the evaluation**: community involvement in evaluation process  **Published:** 2015  **Evaluation period:** 2013-2015  **Sample size:** Not reported  **Age:** Not reported, included youth and adults  **Gender:** 100% male | | |
| Evaluation indicators | Effectiveness | - Reduction in family and domestic violence - Reduction in self-harm and suicide - Wurrumiyanga site: Reduced contact with justice system (50% reduction in the number of men registered with the NT Department of Correctional Services, reduction in rates of recidivism and reoffending) - Women report feeling safer and more supported by the men in their families and communities - Improved social and emotional wellbeing (including enhanced self-esteem and confidence) - Increased re-emergence of cultural celebrations and ceremonies | |
|  | Acceptability | Rates of engagement   - 448 men participated in program activities with many more attending community events and celebrations | |
|  | Feasibility | Enablers   - Co-design by men in the community, ensuring ownership and adaption to local needs, so allowing men to lead the way in their own healing. - Connection to country, culture and identity increase empowerment and confidence to take the cultural lead with younger men, provide increased safety for families and take up employment | |
|  |  | Barriers   - Slow implementation (lack of backing from auspicing bodies) - Lack of human resources (e.g., counsellors, mentors) - Lack of a space for men to meet, cool off and share story and belonging - Limited opportunities for transfer of cultural knowledge from Elders to young men | |
| Other | Funded by the Healing Foundation and the Northern Territory Department of Children and Families | | |

### Table A16. Exemplar program : Red Dust Healing

| Location | National | | |
| --- | --- | --- | --- |
| Setting | Not-for-profit, Aboriginal and Torres Strait Islander owned & designed | | |
| Operational status | In operation since 2007 | | |
| Target population | Aboriginal and Torres Strait Islander men, women and families. Has been adapted for a range of population subgroups: young people in juvenile detention centres, people with disabilities, high school and university students, and community and health workers (e.g., doctors, police, legal practitioners) | | |
| Aim | To address suicide prevention, stolen generations, grief and loss, family and domestic violence, mental health and substance abuse. | | |
| Framework | A holistic, culturally relevant and strengths-based approach | | |
| Guiding principles | - Aboriginal and Torres Strait Islander owned & designed - Culturally appropriate - Holistic: Addresses mental, emotional, spiritual needs and focuses on interconnectedness with family and community - Indigenous Model of Oppression: informed by history and colonization | | - Promoting self-healing, self-determination and empowerment - Solution focused - Flexible and transferable - Provides safe space: programs run on country where possible - Elder involvement - Incorporates reflective practices through evaluations |
| Core components | - Workshops delivered in a relaxed, non-threatening way - Two workshop modalities: 1 day or 3 days - Use of culturally relevant tools or symbolic diagrams: *The tree* (symbolizes different choices individuals can make and the impact of choices on family members); *Bird and Fish* (control, how to deal with grief and loss); *Lore and Law* (difference between L-O-R-E, Land, Origin, Respect, Elders, and L-A-W, Legalities, Attorney General and Westminster system); *JIG syndrome* (Jealousy, Insecurity and Greed); Pouch (solution-based, problem solving concept); *The Mat* (individualized session on a mat), *Suicide Safeguarding* - Post workshop linkage with relevant Aboriginal and Torres Strait Islander services, mentors and at times, buddies. Sometimes follow-up conducted by the facilitator | | |
| Staffing | Founded and facilitated by Warramunga man (Wiradjuri Nation). Additional facilitators are carefully selected and trained by founder/key facilitator.  Service providers and clinicians can be trained to deliver the RDH tools in their practice. | | |
| Evaluation Design | **Design:** Mixed-methods, independent evaluation (Jo Thompson Consulting) (Red Dust Healing, 2018)  **Aboriginal governance, design and execution in the evaluation**: embedded within a participatory action research framework, designed to reflect Aboriginal and Torres Strait Islander worldview whilst respecting histories, cultures and knowledge systems.  **Published:** 2018  **Evaluation period:** Not reported  **Sample size:** 42  **Age:** 33% between ages of 36-45 years old, 33% over the age of 56 years old  **Gender:** 52% female | | |
| Evaluation indicators | Effectiveness | - Improved social and emotional wellbeing sustained after program completion (e.g., increased ability to express deep seated emotions, an improved capacity to deal with grief and loss, greater self-awareness and clarity, the ability to make better choices and consequently changes in their lives, increased skills to bring about conflict resolution in the family and community settings and a stronger sense of cultural and spiritual identity) - Ripple effect: Benefits extend beyond the individual participant to their families, communities, workplaces - Program helped participants reflect on the impact of life choices and decisions on both their own lives and on those around them - The suicide prevention report by the University of Western Australia rate program as having strong effectiveness and good practice (Red Dust Healing, 2018) | |
|  | Acceptability | - Has strong community support (from those who have experienced the benefits of the program in their own lives or recognised profound changes in the lives of family members) - A majority of interviewees reported continued personal use and sharing of RDH tools 2 or more years post program. - Participants report high levels of satisfaction with the program   Rates of engagement   - Since 2007, >15,000 people in almost 300 communities across Australia have completed the program | |
|  | Feasibility | Enablers   - Creation of a culturally, emotionally, and physically safe environment that respects confidentiality - Adaptability and accessibility: program has been replicated in multiple locations, communities and across cultures - Tools employed by the program continue to be used by participants years after completion - Program tools can be delivered by clinicians and service providers - Rapport between founder, facilitators and participants | |
|  |  | Barriers   - Difficult to meet growing demand. Nationwide demand outstrips capacity to fulfill requests - Ongoing support through external services can be difficult to facilitate in remote areas where services are limited | |
| Other | Adapted for use in the Philippines and for Māori populations in New Zealand | | |

### Table A17. Exemplar program: Trauma Assessment, Referral and Rehabilitation Outreach Teams (TARROT) Program (Ngaoara)

| Location | New South Wales, Australian Capital Territory | | |
| --- | --- | --- | --- |
| Setting | Not‐for‐profit, founded by an Aboriginal woman, integrated within schools or ACCHOs | | |
| Operational status | In operation since 2016 | | |
| Target population | Aboriginal and Torres Strait Islander children | | |
| Aim | To provide medical and therapeutic trauma assessment and referral outreach for Aboriginal and Torres Strait Islander children affected by trauma | | |
| Framework | Socio-ecological model, trauma-informed | | |
| Guiding principles | - Child centric - Integrated and multisectoral response - The promotion of positive cultural practices | | - Culturally informed and safe services - Treatment as prevention |
| Core components | - Take referrals from local schools and services to provide regular outreach for children identified with, or suspected of exposure to, the impacts of trauma, violence and abuse - Provision of free culturally informed, sustained outreach specialist assessments - Facilitate case management and coordinate care for at risk and vulnerable children - Promotes connection to culture through cultural activities - Practical support (food, clothing and access to safe shelter) - Outreach with specialist teams - High quality assessment of needs - Care planning - Coordination of care and case management - Capacity development of existing services and professional development of staff | | |
| Staffing | Small teams of multidisciplinary clinical and educational professionals | | |
| Evaluation Design | **Design:** Mixed-methods, independently conducted (Murrawin Consulting, The Burnet Institute) (Ngaoara Limited, 2019)  **Aboriginal governance, design and execution in the evaluation**: Involvement of Murrawin Consulting, an Indigenous consultancy firm  **Published:** 2019  **Evaluation period:** 2016-2019  **Sample size:** 21 interviews (6 staff members, 15 family members); aggregate data for 21 children  **Age:** Not reported  **Gender:** Not reported | | |
| Evaluation indicators | Effectiveness | - Reframed behavioural problems as unresolved trauma, increased the awareness of trauma - Improve social and emotional wellbeing: children feel valued, listened to and cared for - Developed and delivered workforce capacity building (school staff, service staff) - Instilled aspiration and hope - Advocacy for improved services and resourcing to respond to trauma - Creation of a safe space - Improved school engagement - Addressed barriers to accessing specialist services (availability, distance, costs, cultural safety) - Improved quality of care in partner services (e.g., engagement with young people, trauma-informed) | |
|  | Acceptability | - Increase in number of young people engaged in the program - Program highly valued by families and community members   Rates of engagement   - 78 outreach visits completed - 135 children/adolescents referred and engaged with service - 157 specialist assessments completed | |
|  | Feasibility | Enablers   - Employment of local staff members - Filled service delivery gap through the implementation of a comprehensive specialist medical assessment and care planning - Partnership with community - Maintained and unconditional commitment to addressing trauma | |
|  |  | Barriers   - Lack of existing structures to support referrals, screening or ongoing support following assessment | |
| Other | Funded by the Commonwealth Department of Health (Light House Project) | | |

### Table A18. Exemplar service: Waminda - South Coast Women's Health and Welfare Aboriginal Corporation

| Location | New South Wales | | |
| --- | --- | --- | --- |
| Setting | Not-for-profit, primary healthcare, family support and health promotion services, ACCHO | | |
| Operational status | In operation since late 1980s | | |
| Target population | Aboriginal women, children, young people and their families | | |
| Aim | To support Aboriginal women and their families to be strong, independent, prosperous, powerful, and self-determining | | |
| Framework | **Waminda Model of Care (2021), Balaang Healing framework (2021)** | | |
| Guiding principles | **Waminda Model of Care (2021)**   - Self-determination - Physical, emotional, spiritual, mental health and wellbeing - Restoration of power - Decolonisation - Trauma-informed - Strengths-based - Accountability - Collaboration - Leadership - Trauma-informed - Resilience - Culture - Respect - Boldness | | **Balaang Healing framework (2021)**   - Identity and culture - Health and wellbeing - Being - Nyully - Sharing - Ceremony - Song and dance - Belonging and connection - Spirituality - Family and kinship - Nurturing - Language |
| Relevant programs | 1. Nabu Aboriginal family preservation and restoration program 2. Waminda Counselling Services 3. Balaang Healing 4. Case management services 5. Strong Yawa | | |
| Core components | **Nabu Aboriginal family preservation and restoration program:** includes family-led decision making, family yarning sessions, deep listening, critically reflective practices, strengths-based assessments (Growth and Empowerment Measure), liaison with other services (e.g., NDIS, schools, allied health professions, midwives), in-home practical supports, individual and family therapeutic support, mentoring, and coordination of case planning with referral points such as education, housing, health, legal, non-government organisations and justice-related services.  **Waminda Counselling Services:** narrative therapeutic approach for crisis support, advocacy, group work, support for families who are impacted by sexual abuse, family and domestic violence support, interpersonal trauma support, grief counselling. Includes in-house and outreach services.  **Balaang Healing:** Yarning circles, art therapy, Elders groups, Women's groups, Women's gatherings, short term accommodation  **Case management services:** holistic seamless wraparound support for women and their families  **Strong Yawa:** After-hours support (check-in service and crisis hotline)  **Other:** free fresh fruit at the reception desk | | |
| Staffing | **Nabu Aboriginal family preservation and restoration program:** Caseworkers, Family Support Workers, Cultural Mentors, and Elder/Cultural Mentors, managers, counsellors, and intake/administration staff. Partnerships with DCJ and research institutes  **Waminda Counselling Services:** Two Aboriginal Healing Counsellors, one Social and Emotional Wellbeing Counsellor, one Youth Counsellor and a Drug and Alcohol Counsellor  Waminda’s non-Koori staff are included through the Waminda Imperfect Allies group and by participating in cultural inductions and cultural mentoring | | |
| Evaluation Design | No formal, independent evaluations of relevant programs have been published. Evidence of effectiveness, acceptability, enablers and barriers have been extracted from annual reports (Waminda, 2022, 2024), the SNAIC program review (SNAICC, 2021), the Waminda website and blog posts (Armstrong, 2019)  A large evaluation of Waminda’s programs is currently underway, the expected completion date is unknown. | | |
| Evaluation indicators | Effectiveness | - 2021-2022: no children removed, four longer term family cases have been closed - 2016-2017: 100% success rate preservation and 91% for restoration | |
|  | Acceptability | Rates of engagement  **Nabu**   - 2021-2022: 5831 episodes of care; 7716 client contacts - 2023-2024: 54 families supported - Recognised as one of the best Aboriginal-led early intervention models of care in Australia by SNAICC in 2020-2021   **Waminda Counselling Service**   - 2021-2022: 156 clients supported; 292 counselling sessions; 1081 case management sessions; 852 client contacts; 609 episodes of care - 2023-2024: 1877 case management sessions; 557 healing counselling sessions   **Ballang Healing**   - 2021-2022: 315 episodes of care; 359 client contacts - 2023-2024: 440 clients supported   **Strong Yawa**   - 2021-2022: 700 outgoing calls, received 56 crisis calls; 1782 client contacts; 15 staff trained; 1576 episodes of care | |
|  | Feasibility | Enablers:   - **Waminda Counselling Service:** accessible, adaptable, flexible – structured differently to mainstream services (e.g., no restricted number of sessions) - **Nabu:** family buy-in and participation, situated within an ACCHO that places culture and community at its foundation.   Barriers:   - **Balaang Healing:** limited funding - **Waminda Counselling Service:** Long wait-lists due to high demand for counselling services | |

### Table A19. Summary of additional services (n = 65)

| Service/program | Service/program details | Non-specific SEWB/ healing support | Counselling/ therapeutic support | Cultural activities/tools | Yarning circles | Community connection | Referral networks/ service navigation | Psychoeducation | Outreach | Case management | Advocacy | Intake/assessments | Mentoring | Healing space | Residential program | Crisis support hot line |
| --- | --- | --- | --- | --- | --- | --- | --- | --- | --- | --- | --- | --- | --- | --- | --- | --- |
| Akeyulerre | **Name:** Various programs **Location:** NT **Operational?** Yes **Target population:** Individuals, families and communities | ✔️ |  | ✔️ |  | ✔️ |  |  |  |  |  |  |  |  |  |  |
| Armajun Aboriginal Health Service | **Name:** SEWB and Mental Health Service **Location:** NSW **Operational**? Yes **Target population:** Individuals, families and communities | ✔️ | ✔️ |  |  |  | ✔️ | ✔️ |  |  | ✔️ | ✔️ |  |  |  |  |
| Baabayn Aboriginal Corporation | **Name:** Various healing programs **Location:** NSW **Operational?** Yes **Target population:** Individuals, families and communities | ✔️ |  |  |  |  |  |  |  |  |  |  |  |  |  |  |
| Bendigo and District Aboriginal Co-operative | **Name:** Bendigo and District Aboriginal Co-operative Family Safety Programs **Location:** VIC **Operational?** Yes **Target population**: Families | ✔️ | ✔️ |  |  |  |  |  |  | ✔️ |  |  |  |  |  |  |
| Bila Muuji Health services | **Name:** Bila Muuji SEWB Initiative **Location:** NSW **Operational?** Yes **Target population:** Individuals, families and communities | ✔️ | ✔️ |  |  |  |  |  |  | ✔️ |  |  |  |  |  |  |
| Boorndawan Willam Aboriginal Healing Service | **Name:** Men's healing circles **Location:** VIC **Operational?** No **Target population**: Men | ✔️ |  | ✔️ | ✔️ | ✔️ |  |  |  |  |  |  |  |  |  |  |
| Brewarrina Central School and Bourke High School, Healing Foundation | **Name:** School Healing Programs **Location:** NSW **Operational?** Unclear **Target population:** Youth |  |  | ✔️ | ✔️ |  |  |  |  |  |  |  | ✔️ |  |  |  |
| Canberra Rape Crisis Centre | **Name:** The Nguru Program **Location:** ACT **Operational?** Yes **Target population:** Individuals, families and communities |  | ✔️ |  |  |  |  |  |  |  |  |  |  |  |  |  |
| Che-Ki-Dee Pty. Ltd | **Name:** Che-Ki-Dee Cultural Healing Program **Location:** QLD **Operational?** Yes **Target population:** Families | ✔️ |  |  |  | ✔️ | ✔️ |  |  |  |  |  | ✔️ |  |  |  |
| Cherbourg Regional Aboriginal and Islander Community Controlled Health Services | **Name:** SEWB Team & Family Wellbeing Program **Location:** QLD **Operational?** Yes **Target population:** Individuals, families and communities | ✔️ | ✔️ | ✔️ |  | ✔️ | ✔️ |  |  |  |  |  |  |  |  |  |
| Creating a Safe Supporting Environment & Central Australia Aboriginal Congress | **Name:** Aboriginal Australian Relations Program **Location:** National **Operational?** Yes **Target population:** Individuals, families and communities |  |  |  |  |  |  | ✔️ |  |  |  |  |  |  |  |  |
| Department of Communities’ Child Protection and Family Support | **Name:** Stitching Our Futures Together (SOFT) Program **Location:** WA **Operational?** Yes **Target population:** Children | ✔️ |  | ✔️ |  | ✔️ |  | ✔️ |  |  |  |  |  |  |  |  |
| Derbarl Yerrigan Health Service Aboriginal Corporation | **Name:** Weirn Mooditj **Location:** WA **Operational?** Yes **Target population:** Individuals, families and communities |  | ✔️ |  |  |  |  |  |  |  |  |  |  |  |  |  |
| Dhauwurd Wurrung Elderly and Community Health Service | **Name:** Nootyoong Mara Healing Centre  **Location:** VIC **Operational?** Yes **Target population:** Individuals, families and communities | ✔️ |  |  | ✔️ |  |  |  |  |  |  |  |  |  |  |  |
| Dreaming Inside | **Name:** Dreaming Inside **Location:** NSW **Operational?** Yes **Target population:** Men in prison | ✔️ |  |  |  | ✔️ |  |  |  |  |  |  |  |  |  |  |
| Dumbartung Aboriginal Corporation | **Name:** Kootamiara Quab Women's Healing Program **Location:** WA **Operational?** No **Target population:** Women | ✔️ |  | ✔️ | ✔️ |  |  |  |  |  |  |  |  |  |  |  |
| Galangoor Duwalami Primary Healthcare | **Name:** Galangoor Duwalami Primary Healthcare SEWB Program **Location:** QLD **Operational?** Yes **Target population:** Individuals, families and communities | ✔️ | ✔️ |  |  |  | ✔️ | ✔️ |  |  |  |  |  |  |  |  |
| Gallang Place Aboriginal and Torres Strait Islander Corporation Counselling Services | **Name**: Gallang Place **Location:** QLD **Operational?** Yes **Target population:** Youth and Families | ✔️ | ✔️ |  |  |  |  |  |  |  |  |  |  |  |  |  |
| Geraldton Regional Aboriginal Medical Service | **Name:** Geraldton Bringing Them Home Program **Location:** WA **Operational?** Yes **Target population:** Individuals, families and communities |  | ✔️ |  |  |  | ✔️ |  |  |  |  |  |  |  |  |  |
| Geraldton Regional Aboriginal Medical Service | **Name:** Maga Barndi SEWB Support **Location:** WA **Operational?** Yes **Target population:** Families | ✔️ | ✔️ |  |  |  | ✔️ |  | ✔️ |  |  |  |  |  |  | ✔️ |
| Gippsland and East Gippsland Aboriginal Co-operative | **Name:** Sharing Culture, Sharing Knowledge, Healing People **Location:** VIC **Operational?** No **Target population:** Individuals, families and communities |  |  | ✔️ |  | ✔️ |  |  |  |  |  |  |  |  |  |  |
| Gurriny Yealamucka Health Services | **Name:** Family Healing and Wellbeing Services **Location:** QLD **Operational?** Yes **Target population:** Families | ✔️ | ✔️ |  | ✔️ |  |  |  |  |  |  | ✔️ |  |  |  |  |
| Headspace (Inala) | **Name:** United Health Education and Learning Program **Location**: QLD **Operational?** Yes **Target population:** Young people | ✔️ | ✔️ | ✔️ |  |  |  | ✔️ |  |  |  |  |  |  |  |  |
| Healing foundation | **Name:** Young Healers **Location:** National **Operational?** No **Target population:** Youth | ✔️ |  |  |  |  |  |  |  |  |  |  |  |  |  |  |
| Helem Yumba | **Name:** Helem Yumba Central Queensland Healing Centre  **Location:** QLD **Operational?** Yes **Target population:** Individuals, families and communities | ✔️ | ✔️ |  |  |  |  |  |  |  |  |  |  |  |  |  |
| Indigenous Psychological Services | **Name:** Indigenous Psychological Services **Location:** WA **Operational?** Yes **Target population:** Individuals, families and communities | ✔️ | ✔️ |  |  |  |  | ✔️ |  |  |  |  |  |  |  |  |
| Indigenous Wellbeing Centre Ltd | **Name:** Cultural Connect program **Location:** QLD **Operational?** Yes **Target population:** Individuals, families and communities | ✔️ | ✔️ |  | ✔️ |  |  |  |  |  |  |  |  |  |  |  |
| Ironbark Aboriginal Corporation, not-for-profit | **Name:** Ironbark Indigenous Support **Location:** NT **Operational?** Yes **Target population:** Individuals, families and communities | ✔️ |  |  |  |  |  |  |  | ✔️ | ✔️ |  |  |  |  |  |
| Karadi Aboriginal Corporation | **Name:** Karadi Aboriginal Corporation Community Education Training Program **Location:** TAS **Operational?** Yes **Target population:** Families |  |  |  | ✔️ |  |  | ✔️ |  |  |  |  |  |  |  |  |
| Karadi Aboriginal Corporation and Nayri Niara Centre for the Arts of Healing, Healing Foundation | **Name:** Healing our Trauma **Location:** TAS **Operational?** No **Target population:** Individuals, families and communities | ✔️ |  | ✔️ |  |  |  |  |  |  |  |  |  |  |  |  |
| Katherine West Health Board Aboriginal Corporation (KWHB), ACCHO | **Name:** Katherine West Health Board SEWB Program **Location:** NT **Operational?** Yes **Target population:** Individuals, families and communities | ✔️ |  |  |  |  | ✔️ |  |  |  |  |  |  |  |  |  |
| Kinchela Boys Home Aboriginal Corporation | **Name:** Healing program **Location:** NSW **Operational?** Yes **Target population:** Individuals, families and communities | ✔️ | ✔️ | ✔️ | ✔️ | ✔️ |  |  |  |  |  |  |  |  |  |  |
| Larrakia Nation | **Name:** Larrakia Cultural Centre **Location:** NT **Operational?** Yes **Target population:** Individuals, families and communities | ✔️ |  | ✔️ |  | ✔️ |  |  |  |  |  |  |  |  |  |  |
| Lismore Men and Family Centre & Rekindling the Spirit | **Name:** Tweed Yarn Up Group for Indigenous Men  **Location:** NSW **Operational?** Unclear **Target population:** Men |  |  |  | ✔️ |  |  |  |  |  |  |  |  |  |  |  |
| Melaleuca Place | **Name:** Melaleuca Place **Location:** ACT **Operational?** Unclear **Target population:** Youth |  | ✔️ |  |  |  |  |  |  |  |  | ✔️ |  |  |  |  |
| Men’s Outreach Service Aboriginal Corporation | **Name:** Various programs **Location:** WA **Operational?** Yes **Target population:** Men | ✔️ | ✔️ |  |  |  |  |  | ✔️ |  | ✔️ |  |  |  |  |  |
| Men’s Health Group at the Mulungu Medical Centre | **Name:** Adventure-Based Indigenous Therapy **Location:** QLD **Operational?** Yes **Target population:** Young people | ✔️ | ✔️ | ✔️ | ✔️ | ✔️ |  | ✔️ |  |  |  |  | ✔️ |  |  |  |
| Moorundi Aboriginal Community Controlled Health Service | **Name:** Tumbetun Namawi mi:wi Program **Location:** SA **Operational?** Yes **Target population:** Individuals, families and communities | ✔️ | ✔️ | ✔️ | ✔️ | ✔️ | ✔️ |  |  |  |  |  |  |  |  |  |
| Murrigunyah Family and Cultural Healing Centre | **Name:** Healing Our Way Program **Location:** QLD **Operational?** Yes **Target population:** Families | ✔️ |  |  |  |  |  |  |  |  |  |  |  |  |  |  |
| Northern Rivers Community Healing Hub | **Name:** Northern Rivers Community Healing Hub **Location:** NSW **Operational?** Yes **Target population:** Individuals, families and communities | ✔️ | ✔️ | ✔️ | ✔️ | ✔️ |  |  | ✔️ |  |  |  |  | ✔️ |  |  |
| Not reported | **Name:** Family and Community Healing Program **Location:** SA **Operational?** No **Target population:** Families | ✔️ |  | ✔️ |  | ✔️ | ✔️ |  |  |  |  |  | ✔️ |  |  |  |
| Not reported | **Name:** Heal For Life First Nations Healing Service  **Location:** NSW **Operational?** Yes **Target population:** Youth |  |  |  |  |  |  |  |  |  |  |  |  |  | ✔️ |  |
| Nunkuwarrin Yunti of South Australia Inc | **Name:** Various healing programs **Location:** SA **Operational?** Yes **Target population:** Individuals, families and communities | ✔️ | ✔️ |  |  | ✔️ |  |  |  | ✔️ |  |  |  |  |  |  |
| Orana Haven Drug and Alcohol Residential Rehabilitation Service | **Name:** Voluntary rehabilitation program **Location:** NSW **Operational?** Yes **Target population:** Men | ✔️ | ✔️ | ✔️ |  | ✔️ | ✔️ |  | ✔️ | ✔️ |  |  |  | ✔️ | ✔️ |  |
| Ord Valley Aboriginal Health Service | **Name:** Intergenerational Trauma Program **Location:** WA **Operational?** No **Target population:** Youth |  |  | ✔️ |  |  |  |  |  |  |  |  |  |  |  |  |
| Relationships Australia | **Name:** Healing Our Children Program **Location:** NT **Operational?** No **Target population:** Families | ✔️ |  |  |  |  |  |  |  |  |  |  |  |  |  |  |
| Richmond Wellbeing | **Name:** Moorditj Djerpin Wirrin **Location:** WA **Operational?** Yes **Target population:** Individuals, families and communities | ✔️ |  |  |  |  |  |  | ✔️ |  |  |  |  |  |  |  |
| Rumbalara Aboriginal Cooperative | **Name:** Dunguludja Yakapna Traditional Healing Centre **Location:** VIC **Operational?** Yes **Target population:** Families | ✔️ |  |  |  |  |  | ✔️ |  |  |  |  |  |  |  |  |
| Rural and Remote Mental Health Service | **Name:** Deadly Thinking workshop **Location:** Rural and regional Australia **Operational?** Yes **Target population:** Men and women | ✔️ |  | ✔️ | ✔️ | ✔️ |  | ✔️ |  |  |  |  |  |  |  |  |
| Tangentyere Council | **Name:** Various programs **Location**: NT **Operational?** Yes **Target population:** Women and children | ✔️ | ✔️ |  |  |  |  | ✔️ | ✔️ | ✔️ | ✔️ | ✔️ |  |  |  |  |
| Townsville Aboriginal and Islanders Health Services | **Name:** SEWB Services **Location:** QLD **Operational?** Yes **Target population:** Individuals, families and communities | ✔️ | ✔️ |  | ✔️ |  | ✔️ |  |  |  |  | ✔️ |  |  |  |  |
| Trauma-informed Behaviour Support program | **Name:** Trauma-informed Behaviour Support program **Location**: NT **Operational?** Yes **Target population:** Children | ✔️ |  | ✔️ |  |  |  |  |  |  |  | ✔️ |  |  |  |  |
| Turn em around Healing | **Name:** Turn em around Healing **Location:** NT **Operational?** Unclear **Target population:** Youth | ✔️ | ✔️ | ✔️ |  | ✔️ |  |  |  |  |  |  |  | ✔️ |  |  |
| Victorian Aboriginal Community Services Association Limited | **Name:** Various healing programs **Location:** VIC **Operational?** Yes **Target population:** Individuals, families and communities | ✔️ | ✔️ |  |  |  | ✔️ |  | ✔️ | ✔️ |  |  |  |  |  |  |
| Victorian Aboriginal Health Service | **Name:** Her Tribe and His Tribe **Location:** VIC **Operational?** Unclear **Target population:** Men and women | ✔️ |  |  |  | ✔️ |  |  |  |  |  |  |  |  |  |  |
| Victorian Aboriginal Health Service | **Name:** Minajalku Aboriginal Healing Centre **Location:** VIC **Operational?** Yes **Target population:** Individuals, families and communities | ✔️ |  |  | ✔️ |  |  |  |  |  |  |  |  |  |  |  |
| Wakai Waian Healing | **Name:** Wakai Waian Healing **Location:** QLD **Operational?** Yes **Target population:** Individuals, families and communities | ✔️ | ✔️ |  |  | ✔️ | ✔️ |  |  |  |  |  |  |  |  |  |
| Wellington Aboriginal Corporation Health Service (WACHS), ACCHO | **Name:** SEWB Program **Location:** NSW **Operational?** Yes **Target population:** Individuals, families and communities | ✔️ | ✔️ |  |  |  | ✔️ | ✔️ |  | ✔️ |  |  |  |  |  |  |
| Wirraka Maya Health Service Aboriginal Corporation | **Name:** SEWB Programs **Location:** WA **Operational?** Yes **Target population:** Individuals, families and communities | ✔️ | ✔️ | ✔️ | ✔️ |  | ✔️ | ✔️ | ✔️ | ✔️ | ✔️ |  |  |  |  |  |
| Womens Health and Family Services | **Name:** Aboriginal Women's Services **Location:** WA **Operational?** Yes **Target population:** Families | ✔️ |  | ✔️ | ✔️ | ✔️ | ✔️ |  |  |  | ✔️ |  |  |  |  |  |
| Wungening Aboriginal Corporation | **Name:** Wungening Moort **Location:** WA **Operational?** Yes **Target population:** Youth | ✔️ | ✔️ |  |  |  | ✔️ |  |  |  |  |  |  |  |  |  |
| Wurli Wurlinjang Aboriginal Health Service | **Name:** Wurli-Wurlinjang SEWB Unit **Location:** NT **Operational?** Yes **Target population:** Families | ✔️ | ✔️ |  |  |  | ✔️ | ✔️ |  |  | ✔️ |  |  |  |  |  |
| Yawardani Jan-ga (horses helping) | **Name:** The Yawardani Jan-ga project **Location:** WA **Operational?** Yes **Target:** Young people | ✔️ | ✔️ |  |  |  |  |  |  |  |  |  |  |  |  |  |
| Yorgum Healing Services | **Name:** Yorgum Healing Services  **Location:** WA **Operational?** Yes **Target:** Individuals, families and communities | ✔️ | ✔️ | ✔️ | ✔️ |  |  |  |  |  |  |  |  |  |  |  |
| 13YARN | **Name:** 13YARN **Location:** National **Operational?** Yes **Target population:** Individuals, families and communities |  |  |  |  |  |  |  |  |  |  |  |  |  |  | ✔️ |

## Appendix 3: Supplementary results

### **Table A20.** Characteristics of identified services and programs

|  | | **All services** (n = 80) | | **Exemplar services or programs** (n = 15) | |
| --- | --- | --- | --- | --- | --- |
| Outcome | | Count | % | Count | % |
| Relevancy | Trauma specific service for Aboriginal and Torres Strait Islander people | 12 | 15% | 3 | 21% |
|  | Service providing support for Aboriginal and Torres Strait Islander people experiencing traumatic events (family violence, child sexual abuse, Out of Home Care, and natural disasters) | 18 | 23% | 5 | 36% |
|  | Other (general healing services, social and emotional wellbeing) | 30 | 37% | 5 | 29% |
|  | Mix of categories | 20 | 25% | 2 | 14% |
| Quality | 1 | 20 | 25% | 14 | 93% |
|  | Other | 60 | 75% | 1 | 7% |
| Location | ACT | 2 | 3% | 1 | 7% |
|  | NSW | 14 | 18% | 3 | 20% |
|  | NT | 13 | 16% | 4 | 27% |
|  | QLD | 13 | 16% | 1 | 7% |
|  | SA | 5 | 6% | 2 | 13% |
|  | TAS | 2 | 3% | 0 | 0% |
|  | VIC | 12 | 15% | 4 | 27% |
|  | WA | 16 | 20% | 2 | 13% |
|  | National | 5 | 6% | 1 | 7% |
| Operational status | In operation | 63 | 79% | 11 | 73% |
|  | Completed | 11 | 14% | 3 | 20% |
|  | Unclear | 6 | 7% | 1 | 7% |
| Target population | Men and women | 40 | 50% | 5 | 34% |
|  | Families | 14 | 18% | 3 | 20% |
|  | Young people | 14 | 18% | 2 | 13% |
|  | Women and children | 5 | 6% | 3 | 20% |
|  | Men only | 7 | 8% | 2 | 13% |
| Core components | Non-specific SEWB/healing support | 59 | 74% | 6 | 40% |
|  | Counselling/therapeutic support | 43 | 54% | 9 | 60% |
|  | Cultural activities | 38 | 48% | 15 | 100% |
|  | Yarning circles | 27 | 34% | 9 | 60% |
|  | Community connection | 29 | 36% | 10 | 67% |
|  | Referral networks/service navigation support | 30 | 38% | 12 | 80% |
|  | Psychoeducation | 21 | 26% | 9 | 60% |
|  | Outreach | 15 | 19% | 7 | 47% |
|  | Case management/care coordination | 18 | 23% | 9 | 60% |
|  | Mentoring | 10 | 13% | 7 | 47% |
|  | Advocacy | 13 | 16% | 6 | 40% |
|  | Intake/assessments | 9 | 11% | 4 | 27% |
|  | Healing space | 7 | 9% | 4 | 27% |
|  | Crisis support hot line | 4 | 5% | 2 | 13% |
|  | Residential program | 4 | 5% | 2 | 13% |

*Notes*. Some services had more than one location and core component resulting in percentages for these categories totalling more than 100%.

### **Table A21.** Evaluation quality assessment ratings

| Service | Evaluation method | Quality assessment | Reasons for downgrading |
| --- | --- | --- | --- |
| Cultural Healing Programs | Peer review qualitative study | HIGH | Not downgraded |
| Dardi Munwurro | Independent evaluation, cost benefit analysis | MODERATE | Downgraded 1 for study limitations (missing data apparent and unaccounted for) |
| Deadly Connections Community Services (1) | Internal impact report by the organisation, independent social impact/effectiveness evaluation | VERY LOW | Downgraded 1 for study limitations (details not reported) and 1 data accuracy (unclear data analysis plan) |
| Deadly Connections Community Services (2) | Independent | MODERATE | Downgraded 1 for data accuracy (inconsistent data collection) |
| Djirra | Independent, mixed method evaluation | MODERATE | Downgraded 1 for data accuracy (unclear data analysis plan) |
| Kornar Winmil Yunti (KWY) Aboriginal Corporation | Independent, mixed method evaluation | LOW | Downgraded 1 for data accuracy and 1 for indirectness (insufficient data) |
| Marninwarntikura Women's Resource Centre | Independent, mixed method evaluation | LOW | Downgraded 1 for study limitations (details not reported) and 1 data accuracy (unclear data analysis plan) |
| Mpwelarre Health Aboriginal Corporation | Peer reviewed qualitative study | HIGH | Not downgraded |
| Murri School Healing Program | Independent evaluation, cost benefit analysis | MODERATE | Downgraded 1 for study limitations (missing data - 45% response rate to evaluation survey) |
| North Australian Aboriginal Justice Agency Healing Program | Document review | VERY LOW | Downgraded 1 for study limitations (no evaluation plan, data sources unclear, documents used for assessment unavailable) and 1 for data accuracy (subjective measures) |
| Our Men Our Healing Project | Independent, mixed method evaluation | MODERATE | Downgraded 1 for data accuracy (unclear data analysis plan) |
| Red Dust Healing | Independent, mixed method evaluation | HIGH | Not downgraded |
| Trauma Assessment, Referral and Rehabilitation Outreach Teams (TARROT) Program (Ngaoara) | Independent, mixed method evaluation | MODERATE | Downgraded 1 for data accuracy (unclear data analysis plan) |
| Uti Kulin Tjaku Watiku Project (KPYWC) | Independent, mixed method evaluation | MODERATE | Downgraded 1 for data accuracy (unclear data analysis plan) |
| Wadamba Wilam | Internal practice report | VERY LOW | Downgraded 1 for study limitations (details not reported) and 1 data accuracy (unclear data analysis plan) |
| Waminda | No evaluation report/paper | | |

**Appendix references**

Aboriginal Family Violence Prevention And Legal Service Victoria. (2014). *Evaluation Report Of The Aboriginal Family Violence Prevention And Legal Service Victoria’s Early Intervention And Prevention Program*. <https://djirra.org.au/wp-content/uploads/2018/02/SDO-Evaluation-Report-Web-Version-1.pdf>

Armstrong, R. (2019, 31/10/2019). *Strong Women, Strong Culture: Community Control Success Stories at Waminda*. <https://www.ahmrc.org.au/strong-women-strong-culture-community-control-success-stories-at-waminda/>

Bamblett, M., Long, M., Frederico, M., & Salamone, C. (2014). Building an Aboriginal cultural model of therapeutic residential care: The experience of the Victorian Aboriginal Child Care Agency. *Children Australia*, *39*(4), 206-210. <https://doi.org/> doi: 10.1017/cha.2014.28

Black, C., Frederico, M., & Bamblett, M. (2019). Healing through Connection: An Aboriginal Community Designed, Developed and Delivered Cultural Healing Program for Aboriginal Survivors of Institutional Child Sexual Abuse. *The British Journal of Social Work*, *49*(4), 1059-1080. <https://doi.org/10.1093/bjsw/bcz059>

Black, C., Frederico, M., & Bamblett, M. (2024). ‘Healing through culture’: Aboriginal young people's experiences of social and emotional wellbeing impacts of cultural strengthening programs. *Child Abuse & Neglect*, *148*, 106206. <https://doi.org/10.1016/j.chiabu.2023.106206>

Booth, E. (2020). *Deadly Families Evaluation Report* <https://www.parliament.nsw.gov.au/ladocs/other/16432/Deadly%20Connections%20-%20Answers%20to%20questions%20on%20notice%20-%208%20June.pdf#:~:text=Deadly%20Connections%20commissioned%20For-Purpose%20Evaluations%20to%20evaluate%20the,culture%20of%20continuous%20improvement%20and%20clear%20outcomes%20accountability>.

Cahill, M., Brown, R. A., Baker, G., Barnes-Proby, D., & Sandrini, H. (2021). *Australia's Third Action Plan of the National Plan to Reduce Violence Against Women and Their Children, Priority Area 2: Aboriginal and Torres Strait Islander Women and Their Children - Final Report*. <https://www.rand.org/pubs/research_reports/RRA389-1.html>

Carey, T. A. (2013). A qualitative study of a social and emotional well-being service for a remote Indigenous Australian community: implications for access, effectiveness, and sustainability. *BMC Health Services Research*, *13*(1), 1-11. <https://doi.org/> doi: 10.1186/1472-6963-13-80

Chiera, J. (2021). *Wadamba Wilam Practice Approach*. <https://assets.neaminational.org.au/assets/Assets/Services/Wadamba-Wilam/cf9525a81e/Wadamba-Wilam-Practice-Approach-2-FA-WEB.pdf>

Commonwealth of Australia. (2017). *National Strategic Framework for Aboriginal and Torres Strait Islander Peoples’ Mental Health and Social and Emotional Wellbeing 2017-2023*. <https://www.niaa.gov.au/resource-centre/national-strategic-framework-aboriginal-and-torres-strait-islander-peoples-mental>

Deadly Connections. (2021). *Deadly Connections Impact Report 2019-2021*. <https://deadlyconnections.org.au/wp-content/uploads/2022/08/Deadly-Connections-Impact-Report-2019-2021.pdf>

Deloitte Access Economics. (2017). *Cost Benefit Analysis of the Murri School Healing Program*. <https://apo.org.au/sites/default/files/resource-files/2017-09/apo-nid117001.pdf>

Deloitte Access Economics. (2021). *Strengthening Spirit and Culture: A cost-benefit analysis of Dardi Munwurro’s men’s healing programs*. D. A. Economics. <https://www.dardimunwurro.com.au/wp-content/uploads/2021/11/HF_Strengthening_Spirit_and_Culture_Dardi_Munwurro_Report_Oct2021_V5.pdf>

Djirra. (2024). *Annual Report 2023–24*. <https://djirra.org.au/wp-content/uploads/2024/12/Djirra-Annual-Report-2023-24-A4-FA-WEB.pdf>

Gupta, H., Tari-Keresztes, N., Stephens, D., Smith, J. A., Sultan, E., & Lloyd, S. (2020). A scoping review about social and emotional wellbeing programs and services targeting Aboriginal and Torres Strait Islander young people in Australia: Understanding the principles guiding promising practice. *BMC Public Health*, *20*(1), 1-20. <https://doi.org/> doi: 10.1186/s12889-020-09730-1

Hovane, V., Sellers, L., Pickett, K., Mogridge, R., Pickett, L., Wallum, D., & Chan, S. (2023). *Truth-Telling At The Centre: An Evidence-Informed Co-Design Of A Healing Framework For Koorlangka With Complex Trauma*. <https://yorgum.org.au/wp-content/uploads/Community-Report_Truth-Telling-at-the-Centre_Healing-Framework-for-Koorlangka-with-Complex-Trauma.pdf>

Marninwarntikura Women's Resource Centre. (2021). *2021 Annual Report*. <https://cdn.shopify.com/s/files/1/1613/1919/files/Marnin_Annual_Report_2021_Web.pdf?v=1643849165>

Marninwarntikura Women's Resource Centre. (2022). *Marninwarntikura Healing Framework*. <https://cdn.shopify.com/s/files/1/1613/1919/files/Marnin_Healing_Framework_Report_2023_WEB_A4.pdf?v=1681741563>

Marninwarntikura Women's Resource Centre. (2023). *2023 Annual Report*. <https://cdn.shopify.com/s/files/1/1613/1919/files/Marnin_Annual_Report_2023_FINAL_Web_A4.pdf?v=1711333777>

Marninwarntikura Women's Resource Centre. (2024). *2024 Annual Report* <https://cdn.shopify.com/s/files/1/1613/1919/files/Marnin_Annual_Report_2024_FINAL_Web_A4.pdf?v=1731887812>

Ngaoara Limited. (2019). *Trauma Assessment, Referral and Rehabilitation Outreach Teams (TARROT): A description of the program and an evaluation of its impact. A report to the Department of Health*. <https://treasury.gov.au/sites/default/files/2020-09/115786_NGAOARA_LIMITED_-_SUPPORTING_DOCUMENTATION.pdf>

Pearce, J. (2017). *Marninwarntikura Women’s Shelter Review Stage 2 Report*. <https://cdn.shopify.com/s/files/1/1613/1919/files/Marninwarntikura_Shelter_review_-_Stage_2_-_2017.pdf?6162287972779629518>

Prince, J. (2021). *Intergenerational trauma initiative: North Australian Aboriginal Justice Agency Healing Program review. Understanding the impact*. <https://healingfoundation.org.au/app/uploads/2021/09/HF_NAAJA_Intergenerational_Trauma_Initiative_Final_Report_Jul2021_V2_WEB.pdf>

Red Dust Healing. (2018). *Red Dust Healing Program Evaluation* <https://www.thereddust.com/index.php/download_file/force/66/177>

SNAICC. (2021). *Waminda – South Coast Women’s Health And Welfare Aboriginal Corporation: Nabu Aboriginal family preservation and restoration program*. SNAICC. <https://www.snaicc.org.au/wp-content/uploads/2022/02/SNAICC-Early-Intervention-WAMINDA-final.pdf>

The Healing Foundation. (2015). *Our Men Our Healing: Evaluation Report*. <https://healingfoundation.org.au/app/uploads/2017/03/OMOH-60-pg-report-small-SCREEN-singles.pdf>

The Healing Foundation. (2019). *A Theory of Change for Healing*. <https://cdn.healingfoundation.org.au/app/uploads/2019/04/18122127/HF_Theory_of_Change_A4_Mar2019_WEB.pdf>

The Healing Foundation. (2022). *Stolen Generations Collective Healing Initiatives Rounds 1–6: impacts and findings*. <https://healingfoundation.org.au/app/uploads/2022/12/Stolen_Generations_Collective_Healing_Rounds_1-6_Jun22_V5.pdf>

Togni, S. (2018). *Uti Kulintjaku Project: Summary Report*. <https://www.npywc.org.au/wp-content/uploads/3.-Evaluation-Summary-Report_Uti-Kulintjaku-Project_Oct-2018_FINAL.pdf>

Togni, S. (2019). *Uti Kulintjaku Watiku Project: 2019 Evaluation Report*. <https://www.npywc.org.au/wp-content/uploads/Evaluation-Report_UK-Mens-Project_Sept-2019_FINAL.pdf>

Togni, S. J. (2017). The Uti Kulintjaku Project: The path to clear thinking. An evaluation of an innovative, Aboriginal‐led approach to developing bi‐cultural understanding of mental health and wellbeing. *Australian Psychologist*, *52*(4), 268-279. <https://doi.org/10.1111/ap.12243>

Waminda. (2022). *Waminda Annual Report 2021 - 2022*. <https://drive.google.com/file/d/1efyeqH4sawWAe0tcV7cu-0boECXMOYJu/view>

Waminda. (2024). *Waminda Annual Report 2023 - 2024*. <https://waminda.org.au/resources/>

Wise, S., Jones, A., Johnson, G., Croisdale, S., Callope, C., & Chamberlain, C. (2024). Healing and wellbeing outcomes of services for Aboriginal people based on cultural therapeutic ways: A systematic scoping review. *American journal of community psychology*, *74*(1-2), 5-15. <https://doi.org/10.1002/ajcp.12759>
